# MiR-1287-5p inhibits triple negative breast cancer growth by interaction with phosphoinositide 3-kinase CB, thereby sensitizing cells for PI3Kinase inhibitors

**DOI:** 10.1186/s13058-019-1104-5

**Published:** 2019-02-01

**Authors:** Daniela Schwarzenbacher, Christiane Klec, Barbara Pasculli, Stefanie Cerk, Beate Rinner, Michael Karbiener, Cristina Ivan, Raffaela Barbano, Hui Ling, Annika Wulf-Goldenberg, Stefanie Stanzer, Gabriel Rinnerthaler, Herbert Stoeger, Thomas Bauernhofer, Johannes Haybaeck, Gerald Hoefler, Stephan Wenzel Jahn, Paola Parrella, George Adrian Calin, Martin Pichler

**Affiliations:** 1 Division of Oncology, Department of Internal Medicine, Medical University of Graz (MUG), Graz, Austria; 2 Research Unit for Non-coding RNAs and Genome Editing, Medical University of Graz (MUG), Graz, Austria; 3 Fondazione IRCCS Casa Sollievo della Sofferenza Laboratorio di Oncologia, San Giovanni Rotundo, FG Italy; 4 Biomedical Research, Medical University of Graz, Graz, Austria; 5 Department of Phoniatrics, ENT University Hospital, Medical University of Graz, Graz, Austria; 6 Department of Experimental Therapeutics -- Unit 1950, The University of Texas MD Anderson Cancer Center, Houston, TX USA; 7 The Center for RNA Interference and Non-coding RNAs, The University of Texas, MD Anderson Cancer Center, Houston, TX USA; 8 Experimental Pharmacology & Oncology GmbH, EPO, Berlin, Germany; 9 IIIrd Medical Department with Hematology and Medical Oncology, Hemostaseology, Rheumatology and Infectious Diseases, Oncologic Center, Paracelsus Medical University, Salzburg, Austria; 10 Institute of Pathology, Medical University of Graz, Graz, Austria; 11 Department of Pathology, Medical Faculty, Otto-von-Guericke University Magdeburg, Magdeburg, Germany

**Keywords:** Non-coding RNA, microRNAs, Breast cancer

## Abstract

**Background:**

Non-coding RNAs and especially microRNAs have been discovered to act as master regulators of cancer initiation and progression. The aim of our study was to discover and characterize the function of yet functionally uncharacterized microRNAs in human breast carcinogenesis.

**Methods:**

In an unbiased approach, we utilized an established model system for breast cancer (BC) stem cell formation (“mammosphere assay”) to identify whole miRNome alterations in breast carcinogenesis. Clinical samples of BC patients were used to evaluate the human relevance of the newly identified miRNA candidates. One promising candidate, miR-1287-5p, was further explored on its impact on several hallmarks of cancer. The molecular mode of action was characterized by whole transcriptome analysis, in silico prediction tools, miRNA-interaction assays, pheno-copy assays, and drug sensitivity assays.

**Results:**

Among several other microRNAs, miR-1287-5p was significantly downregulated in mammospheres and human BC tissue compared to normal breast tissue (*p* < 0.0001). Low expression levels were significantly associated with poor prognosis in BC patients. MiR-1287-5p significantly decreased cellular growth, cells in S phase of cell cycle, anchorage-independent growth, and tumor formation in vivo. In addition, we identified PIK3CB as a direct molecular interactor of miR-1287-5p and a novel prognostic factor in BC. Finally, PI3Kinase pathway chemical inhibitors combined with miR-1287-5p mimic increased the pharmacological growth inhibitory potential in triple negative BC cells.

**Conclusion:**

Our data identified for the first time the involvement of miR-1287-5p in human BC and suggest a potential for therapeutic interventions in difficult to treat triple negative BC.

**Electronic supplementary material:**

The online version of this article (10.1186/s13058-019-1104-5) contains supplementary material, which is available to authorized users.

## Translational relevance

Triple negative breast cancer is an aggressive variant with limited treatment options due to a lack of understanding of molecular and genetic characteristics. MicroRNAs have been described as master regulators of carcinogenesis in all types of cancer of epithelial origin, and clinical trials using microRNA-based therapeutics have entered the stage of in-human trials. The fundamental understanding which and how microRNAs are relevant in triple negative breast cancer may improve the understanding of pathogenesis and carry potential for novel treatment options. In this study, we add evidence that one of these microRNAs, miR-1287-5p, is downregulated in mammospheres and breast cancer tissues, and these low expression levels are associated with poor survival in breast cancer patients. Based on these clinically relevant observations, we comprehensively characterized miR-1287-5p in cell lines and mice and identified this microRNA for the first time as a tumor suppressive factor in triple negative breast cancer. Moreover, miR-1287-5p directly interacts with members of the PI3Kinase signaling pathway and sensitizes cells to PI3Kinase inhibitors. Our study provides for the first time evidence that miR-1287-5p contributes to breast carcinogenesis and delivery of miR-1287-5p together with PI3Kinase inhibitors which might represent a novel treatment strategy in triple negative breast cancer patients.

## Background

Breast cancer (BC) accounted for nearly 30% of all new cancer diagnoses in women in 2017 in the Unites States [1]. BC is a heterogeneous disease in terms of clinical outcome, biological behavior, and treatment response [2]. BC can be categorized by genomic expression patterns into several subtypes including luminal A, luminal B, human epidermal growth factor receptor type 2 (HER2) enriched, basal-like, claudin-low, and normal-like subtype [3, 4]. Many studies reported that women with basal-like BC had shorter relapse-free and overall survival times than women with other types of BC [5]. The basal-like BC type has many overlapping features of the so-called triple negative breast cancer (TNBC), a category commonly defined by the routinely used immunohistochemistry workflow [5]. TNBC is characterized by the absence of estrogen receptor (ER-) and progesterone receptor (PR-) together with a lack of HER2 receptor expression. Patients with TNBC have a poor prognosis with short disease-free and overall survival and an increased risk for early relapse or distant recurrence within the first years after initial diagnosis [6]. Because of the lack of understanding the biology and the lack of identification of new druggable targets, there are only few molecular-targeted therapies available for TNBC including recently established PARP inhibitors in BRCA1-mutated tumors [7]. Thus, search for novel molecular factors that influence growth and carcinogenesis of TNBC cells is an unmet medical need for developing novel strategies to prevent cancer progression and BC-related death.

MicroRNAs (miRNAs) are a class of small non-coding RNAs that negatively regulate protein-coding gene expression through post-transcriptionally targeting of their respective mRNAs [8]. These small endogenous RNA molecules, approximately 22 nucleotides in length, are evolutionarily ubiquitous, indicating their participation in a wide range of genetic regulatory and immune-related pathways [9–12]. It has been proven that miRNAs are involved in cancer pathogenesis of solid tumors and can act either dominantly or recessively, through regulation of formation of proteins [13, 14]. MiRNAs might also serve as promising biomarkers for diagnosis, prognosis, and prediction of treatment efficacy in BC and other types of cancer [15–18]. The so-called mammosphere assay has been developed by Dontu and colleagues and is an in vitro cell culture model for human mammary epithelial cells that are growing as tumor spheres under non-adherent conditions [19]. These mammospheres are multicellular, three-dimensional structures, and they contain a higher number of mammary stem cells and undifferentiated progenitor cells [19]. The cancer stem cell (CSC) theory suggests that CSCs or cells with stemness features are an important factor in carcinogenesis, tumor growth, metastasis, and recurrence of cancer because of their tumor-initiating and tumor maintenance ability [20]. In the present study, we aimed to identify novel miRNAs with a role in BC carcinogenesis based on the enrichment in this stem cell-related mammospheres. Based on a global microRNome-based profiling and unbiased approach, we further deeply explored one yet uncharacterized miRNA, miRNA-1287-5p, in TNBC carcinogenesis.

## Materials and methods

### Cell culture

In this study, we used the triple negative BC cell lines SUM159, BT549, MDA-MB-231, MDA-MB-468, and HCC1937; the endocrine receptor positive T47D, MCF7, and BT474; and the HER2 receptor positive HCC1937 and SKBR3 [20]. The BC cell line SUM159 was purchased from Asterand (Detroid, MI, USA); KPL-1 was from DMSZ (Braunschweig, Germany); BT549, MDA-MB-231, MDA-MB-468, MCF-7, BT474, T47D, HCC1419, HCC1937, SKBR3, and the embryonic kidney cell line HEK-293 were obtained from American Type Culture Collection (ATCC; Manassas, CA, USA). BT549, T-47D, HCC-1419, and HCC1937 cells were maintained in RPMI 1640 (with l-glutamine, Gibco, Darmstadt, Germany) 10% fetal bovine serum (FBS) gold (Biochrom, Cambridge, UK) and 1% penicillin/streptomycin (for all used cell lines: penicillin: 10000 units/ml, streptomycin: 10.000 μg/ml, Gibco); SUM159 cells were grown in Ham’s F12 containing 1 mmol/L l-glutamine (GE Health Care Life Sciences, Pittsburgh, USA), 2 mmol/L HEPES buffer (Gibco), 5 μg/ml insulin actrapid (Novo Nordisk, Vienna, Austria), 1 μg/ml hydrocortisone (Sigma-Aldrich, Vienna, Austria), 1% penicillin/streptomycin (Gibco), and 5% FBS gold (Biochrom). MDA-MB-231, MDA-MB-468, KPL-1, and HEK-293 cells were maintained in high-glucose DMEM (Gibco), 10% FBS gold (Biochrom), and 1% penicillin/streptomycin (Gibco). MCF-7 cells were grown in MEM with Earle’s salts containing 2 mmol/L l-glutamine (PAA, Pasching, Austria), 1% sodium pyruvate (Gibco), 1% penicillin/streptomycin (Gibco), and 10% FBS gold (Biochrom). BT474 were cultured in RPMI 1640 (with l-glutamine, Gibco), 20% FBS gold (Biochrom), 1% penicillin/streptomycin (Gibco), and 10 μg/ml insulin Actrapid (Novo Nordisk). SKBR3 were grown in McCoy’s 5A modified Medium (Gibco; w/o l-glutamine, 2,2 g/L sodium bicarbonate) 1% penicillin/streptomycin (Gibco) and 10% FBS gold (Biochrom). All cell lines were grown in a 5% CO_2_ humidified incubator at 37 °C. The BC cell lines were authenticated at the Cell bank of the Core Facility of the Medical University of Graz, Austria, by performing a STR profiling analysis (Kit: Promega, PowerPlex 16HS System; Cat.No. DC2101, last date of testing: March 3, 2016). Mycoplasma testing was performed using the Venor GeM Mycoplasma Detection Kit (Minerva Biolabs, Berlin, Germany). After obtaining a confluence of approximately 70%, total RNA was isolated following a standard TRIzol protocol (Invitrogen, Thermo Fisher Scientific, Waltham, MA USA) according to the manufacturer’s instructions.

### Sphere assay (“Mammosphere assay”)

To identify novel miRNAs associated in breast carcinogenesis, we generated tumor spheres as previously described with slight modifications [19]. In detail, the adherent growing BC cell lines were dissociated into single cells using trypsin/EDTA and 2,000 single cells per well seeded in ultra-low attachment six-well plates (Corning, NY, USA) using serum-free MEBM (Lonza, Basel, Switzerland) medium (SFM) supplemented with 1xB27 supplement (Gibco), 20 ng/ml human epidermal growth factor (EGF; Peprotech, Hamburg, Germany), 10 ng/ml human basic fibroblast growth factor (FGF; Peprotech), 20 IU/ml Heparin (Baxter, Vienna, Austria), and 1% antibiotic/antimycotic solution (Thermo Fisher Scientific, containing 10,000 units/mL of penicillin, 10,000 μg/mL of streptomycin, and 25 μg/mL of Gibco Amphotericin B). Spheres and corresponding adherent growing cells were harvested and RNA was extracted for the microarray analysis using the miRNeasy Kit (Qiagen, Hilden, Germany).

### MiRNA microarray analysis

To assess differentially expressed miRNAs in adherent growing cells compared to mammospheres in three different BC cell lines in biological triplicates, total RNA was isolated using the miRNeasy Mini Kit (Qiagen, Hilden, Germany) according to the manufacturer’s instructions. Total RNA was checked on Bioanalyzer BA2100 (Agilent; Foster City, CA) for excellent quality. All samples showed a RIN (RNA integrity number) > 9. The whole transcriptome analysis was performed on Affymetrix GeneChip miRNA Arrays v3 (Affymetrix; Santa Clara, CA, USA) as instructed by the manufacturer’s protocol. Hybridizations were done at the Core Facility Molecular Biology at the Centre of Medical Research at the Medical University of Graz. Raw data is available at the Gene Expression Omnibus (GEO; accession number GSE103218). Heat map for the miRNA expression levels was generated by R software.

### Patient cohort/clinical data

For comparison of matched normal breast and cancer tissue samples, a cohort of 131 BC patients was provided by the Laboratory of Oncology, IRCCS Casa Sollievo della Sofferenza, Viale Padre Pio, 71013 San Giovanni Rotondo, FG, Italy. Ethical approval and informed consent were obtained to fulfill the institutional requirements. RNA preparation and quantitative PCR were performed as previously described [21]. The relative expression levels of miR-1287-5p were determined by qRT-PCR, and expression differences in cancer tissue were compared against normal tissue. To perform a confirmation in a second cohort, we downloaded and analyzed data of publicly available patients from the Cancer Genome Atlas Project (TCGA; https://cancergenome.nih.gov/) for BC patients (Download date: December 2018). In detail, level 3 Illumina miRNASeq (Illumina Sequencing technology: Genome Analyzer) were used for miRNA expression analysis. We derived the “reads_per_million_miRNA_mapped” values for mature forms for each miRNA from the “isoform_quantification” files. We downloaded patient clinical information for the TCGA patients with breast invasive carcinoma (BRCA) from cbioPortal (http://www.cbioportal.org/). For the miRNA-Seq data for primary tumors, we derived the ‘reads_per_million_miRNA_mapped’ values for the mature form of hsa-miR-1287-5pMIMAT0005878 hsa-miR-4521 MIMAT0019058 hsa-miR-27a-5p MIMAT0004501 hsa-miR-3150b-3p from the “Isoform Expression Quantification” files from Genomic Data Commons Data Portal (https://portal.gdc.cancer.gov/). The log2-transformation was applied to miRNASeq data. We ended up with a number of 916 primary tumor cases and 93 normal adjacent tissue with miRNA data. Among them there are 92 matched tumor-normal pairs. To be able to determine the expression difference for each miRNA between normal and tumor tissue, we first employed a Shapiro-Wilk test to verify if the data follows a normal distribution. Accordingly, the *t*-test, respectively the nonparametric Mann Whitney Wilcoxon test, was applied to assess the relationship between miRNA expression and tissue type. We performed paired and unpaired comparisons. A box-and-whisker plot (Box plot represents first (lower bound) and third (upper bound) quartiles, whiskers represent 1.5 times the interquartile range) was used to visualize the data. Analyses were carried out in R statistical environment (version 3.4.1) (http://www.r-project.org/). All tests were two-sided and considered statistical significant at the 0.05 level. For testing the prognostic significance of miR-1287-5p, we made use of the publicly available online tool (http://kmplot.com/analysis/index.php?p=service&cancer=breast_mirna) to analyze 1262 patient data from different cohorts [22]. For analysis of the prognostic value of PI3KCB, we used two publicly available online tools (http://www.oncolnc.org/ for the TCGA data set), and for validation cohort, the Kaplan-Meier plotter (http://kmplot.com/analysis/index.php?p=service&cancer=breast) as previously reported [23].

### Quantitative RT-PCR for miRNAs and mRNAs

For quantification of miRNA levels in BC cell lines, 1 μg of total RNA was reverse transcribed by the miScript II RT Kit (Qiagen) according to the manufacturer’s protocol. The following miScript Primer Assays (Qiagen) were used to validate miR-1287-5p in adherent growing cells compared to mammospheres from the miRNA microarray: Hs_miR-1287_1 miScript Primer Assay and Hs_RNU6-2_1 miScript Primer Assay. The miScript Primer Assays were applied on a LightCycler 480 Real-Time PCR System (Roche Diagnostics, Mannheim, Germany) using the miScript SYBR Green PCR Kit (Qiagen) according to the protocol. Measurements were carried out in technical and biological triplicates and RNU6-2 was used as a housekeeper. Relative miRNA expression levels were calculated using the 2^−ΔΔCT^ method according to Livak and Schmittgen [24].

For detection of mRNA expression levels, 1 μg of total RNA was reverse transcribed by using QuantiTect Reverse Transcription Kit (Qiagen) according to the manufacturer’s protocol. Quantitative RT-PCR was carried out in technical duplicates of biological triplicates using primers specific for *PIK3CB*, *LAYN*, *RAP2B*, *SMAD2*, *PLD5*, *CORO2A*, *GAPDH*, and *U6*. Primer sequences are listed in Additional file 1: Table S1. Quantitative RT-PCR was done on a LightCycler® 480 Real-Time PCR System (Roche Diagnostics) using the QuantiTect SYBR Green PCR Kit (Qiagen) according to the manufacturer’s standard protocol. The arithmetic mean of the housekeeping genes GAPDH and U6 was used for normalization, and relative gene expression levels were calculated using a standard 2^−ΔΔCT^ method [24]. Each experiment was performed in three independent biological replicates.

### Protein extraction and Western blot

Total proteins from BC cells were extracted using radioimmunoprecipitation assay (RIPA) buffer (Sigma-Aldrich). Of total cellular proteins, 20 μg were resuspended in 4x Laemmli buffer (BioRad, Hercules, CA, USA) and heated at 95 °C for 10 min. Proteins were separated by a 4–15% Mini-PROTEAN® TGX™Precast Gel (BioRad), transferred onto a nitrocellulose membrane (BioRad), and the membrane was blocked for 1 h with 3% non-fat dry milk in 1xTris buffered saline (TBS; BioRad)/0.1% Tween-20 (Sigma-Aldrich). Immunoblotting was performed, and antibodies specific for PIK3CB (mAb #3011, CellSignaling, diluted 1:1000 in 1% non-fat dry milk in Tris buffered Saline/0.1% Tween-20) and β-actin (AC-15, Sigma-Aldrich, diluted 1:5000 in 1% non-fat dry milk in Tris buffered Saline/0.1% Tween-20) were detected using HRP-conjugated anti-mouse (Dako, Glostrup, Denmark, dilution 1:5000) or anti-rabbit (Santa Cruz, dilution 1:1000) antibodies. Visualization was performed using an enhanced chemiluminescence detection system (Super Signal West Pico, Thermo Scientific, Rockford, IL) on a BioRad ChemiDoc Touch device. Relative quantification of protein expression was performed using the ImageJ (NIH, Bethesda, Maryland) software. Therefore, the band density of the protein of interest was measured and divided by the density of the loading control beta actin.

### In vitro transient transfection of miR-1287-5p mimic/inhibitor

To achieve transient overexpression or reduction of miR-1287-5p expression in BC cell lines, the miR1287-5p mimic (Syn-hsa-miR-1287-5p miScript miRNA Mimic, 50 nM), inhibitor (Anti-hsa-miR-1287-5p miScript miRNA Inhibitor, 50 nM), and recommended negative control (miScript Inhibitor Negative Control and AllStars Negative Control, 50 nM) were used according to protocol recommendation of the manufacturer (Qiagen). Cells in 6-well plates were transfected using the fast-forward transfection protocol; cells in 96-well plates were transfected using the reverse transfection protocol using the HiPerFect Transfection Reagent protocol (Qiagen) according to the manufacturer’s instructions. To confirm the reached levels of overexpression or silencing, quantitative RT-PCR was used in comparison to the respective controls.

### Lentiviral-transduced stable overexpression of miR-1287 precursor and mature form of miR-1287-5p

MDA-MB-231 and SUM159 cells were seeded and incubated overnight in complete growth medium in 24-well plates. After 24 h, the medium was replaced with complete growth medium containing ViralPlus Transduction Enhancer (1:200, ABM, Richmond, BC, Canada) and 8 μg/ml polybrene (Santa Cruz Biotechnology, Santa Cruz, CA, USA). Cells were infected by adding 10 μl miR-1287 precursor (LentimiRa-GFP-hsa-miR-1287precursor Virus, ABM), miR-1287-5p lentiviral particles (LentimiRa-GFP-hsa-miR-1287-5p Virus, ABM), or control lentiviral particles (Lenti-III-mir-GFP Control Virus ABM). Stably transfected cells were continuously selected with 1 μg/ml puromycin dihydrochloride (Gibco) for 4 weeks and expression levels were determined by qRT-PCR.

### Cellular growth assays

To test whether manipulation of miR-1287-5p expression levels influences cellular growth rate of BC cells, we measured the short-term effects (96 h) by applying the WST-1 proliferation assay (Roche, Applied Science, Mannheim, Germany). Cell lines were seeded in 96-well plates and transiently transfected with the mimic or inhibitor using the reverse transfection protocol and HiPerFect Transfection Reagent (Qiagen) in six technical replicates. Stable lentiviral-transduced cells were also seeded in 96-well plates. Cells were incubated from 24 to 96 h, and every 24 h, the WST-1 proliferation reagent was added in the wells according to the manufacturer’s recommendations. Colorimetric changes were measured using a SpectraMax Plus (Molecular Devices, Germany) at a wavelength of 450 nm with a reference wavelength at 620 nm. Three independent biological replicates were performed each.

To confirm the cellular growth changes after altered miR-1287-5p expression levels by a second independent method, a clonogenic (i.e., colony formation assay) was performed. Transient transfected cells were trypsinized 24 h after transfection. After trypsinization, cells were counted and seeded for colony formation assay in six-well plates at 100–500 cells/well, depending on the cell line. Cells were cultured at 37 °C and 5% CO_2_ and after 10–21 days, cells were fixed as well as stained with 0.01% (*w*/*v*) crystal violet (Sigma-Aldrich) in 20% methanol and PBS. The number of colonies was counted, and each experiment was carried out in biological and technical triplicates.

### Xenograft experiments

To measure the effects of miR-1287-5p in vivo, we injected SUM159 cells stably overexpressing miR-1287-5p or control transduced cells into 5-week-old female nude mice (NU/NU Crl:NU-Fox1nu, Charles River Laboratories; Sulzfeld, Germany). Briefly, 1 × 10^6^ cells were resuspended in phosphate-buffered saline (PBS; 1:1 mixed with matrigel, Corning) and subcutaneously injected into the mammary fat pad of the mice. Cells with miR-1287-5p overexpression were injected in the left mammary fat pad, control cells in the right. A total number of seven mice were used for the in vivo experiments. Tumor growth was monitored every few days by caliper measurements, and animals were sacrificed before tumors reached a diameter of 10 mm. Tumors were harvested for histological analyses, and tumor volumes were calculated by the equation *V*(mm^3^) = (width)^2^ × length/2. All animal work was done in accordance with a protocol approved by the Institutional Animal Care and Use Committee at the Austrian Federal Ministry for Science and Research (BMWF) (BMWFW-66.010/0046-WF/V/3b/2016). For experiments with the miR-1287 precursor overexpressing cells, xenografts were independently generated by an external company. This experiment has been performed by a commercially external facility (EPO Berlin-Buch GmbH, Berlin, Germany) which was completely blinded to the other results of this study. 1 × 10^6^ SUM159 cells were injected subcutanously in mammary fad-pad in a volume of 20 μl to NMRI:nu/nu mice (Janvier Labs, Paris, France). Per group, seven mice were inoculated with cells. All animal experiments were performed under the guidelines of the German Animal Protection Law and with approval by the local responsible authorities. Mice were observed daily for their health status. Mice were sacrificed after 4 weeks after cell inoculation, and the size of tumors relative to the start volume was measured.

### Caspase 3/7 assay

Caspase-Glo 3/7 assay (Promega, Madison, WI, USA) was applied to measure the activity of caspase 3 and 7 according to the manufacturer’s instructions. For transient transfection experiments, BC cell lines were seeded in 96-well plates and transfected using the reverse transfection protocol and HiPerFect Transfection Reagent (Qiagen) in five technical replicates. Caspase 3 and 7 activity was measured 48 h after transient transfection. Lentiviral stable-transduced cells were also seeded in 96-well plates, and apoptosis was measured after 48 h. After adding the substrate, luminescence was measured using a luminometer (LumiStar, BMG Labtech, Ortenberg, Germany).

### Flow cytometric analysis of cell cycle with propidium iodide DNA staining

A number of 100,000 cells were seeded and transfected using the fast-forward protocol (Qiagen) in six-well plates and after 48-h cell cycle analysis was performed. Cells were fixed in 75% ethanol overnight at 4 °C, resuspended in 0.2% FBS/PBS, RNAse A treated (Qiagen, 100 μg/mL), stained with propidium iodide (PI; Sigma-Aldrich) at a final concentration of 40 μg/mL, and analyzed by flow cytometry at a BD LSRII Flow Cytometer. All analyses were performed in triplicates, and 10,000 gated events per sample were counted.

### Soft agar assay

The efficiency of colony formation of cells with altered miR-1287-5p expression in soft agar was determined by plating 2500 cells in complete growth medium containing 0.35% low gelling temperature agarose (Sigma-Aldrich) over 2 ml of growth medium containing 0.5% agar (Sigma-Aldrich) in a 35-mm dish. Cells were cultured at 37 °C and 5% CO_2_ for up to 4 weeks. Colonies were stained with 0.005% crystal violet (Sigma-Aldrich) in 20% methanol, and the number of colonies was counted using a microscope.

### In vitro transient siRNA transfection

BC cell lines were transiently transfected with a short-interfering siRNA for PIK3CB (phosphatidylinositol-4,5-bisphosphate 3-kinase catalytic subunit beta) (Hs_PIK3CB_5, Qiagen, 20 nM) to knockdown the gene of interest using the fast-forward transfection method in six-well plates and the reverse transfection method in 96-well plates according to the HiPerFect Transfection Reagent protocol (Qiagen). All Stars Negative Control siRNA (Qiagen, 20 nM) was used as negative control; AllStars Cell Death Control siRNA (Qiagen, 20 nM) was used to confirm transfection efficiency.

### MiR-1287-5p target identification

To detect putative miR-1287-5p target mRNAs, we applied mRNA microarrays in stable miR-1287 overexpressing SUM159 cells. Total RNA from lentiviral-transduced SUM159 miR-1287-5p overexpressing cells versus control cells in biological triplicates was isolated using the miRNeasy Mini Kit (Qiagen; Hilden, Germany; Cat No. 217004) according to the manual. The whole transcriptome analysis was performed on Affymetrix Human Gene 2.0 ST mRNA Arrays (Affymetrix; Santa Clara, CA, USA) as previously described. In detail, 250 ng of the total RNA was amplified with Affymetrix WT PLUS Reagent Kit (Affymetrix; Santa Clara, CA; Cat No. 703147) as instructed by the manufacturer’s protocol. Additionally, the cDNA was quality checked on the BioAnalyzer BA2100 (Agilent, Foster City, CA) using the RNA 6000 Nano LabChip (Agilent; Foster City, CA; Cat.No. 5065-4476). An examination of ~ 250 ng generated cRNA showed a fragment size > 2000 nt which was satisfying for further processing. The hybridization cocktail was prepared as suggested by the manual and hybridized on the arrays for 18 h at 45 °C while rotating in the hybridization oven. Washing and staining (GeneChip® HT hybridization, Wash and Stain Kit; Affymetrix, Santa Clara, CA; Cat No. 900720) was done with the Affymetrix Genechip® fluidics station 450 according to the manual (protocol on fluidics station: FS450_0002). Arrays were scanned with the Affymetrix GeneChip scanner GCS3000.

The evaluation of the hybridization controls and pre-analysis was done with Affymetrix Expression Console EC 1.3.1. Hybridizations were done at the Division Core Facility Molecular Biology at the Centre of Medical Research at the Medical University of Graz. Data pre-processing and filtering was performed using Partek Genomics Suite, v.6.6 (RMA (background correction, quantile normalization across all chips in the experiment, log2 transformation, median polish summarization)). Raw data are available at the Gene Expression Omnibus (accession number GSE103388).

### In silico target prediction

We performed a bioinformatics target prediction based on global gene expression analysis and comprising to distinct approaches as previously described [25, 26]. Gene expression data was filtered for transcripts that were at least 2-fold downregulated by miR-1287-5p (versus control cells). This subset was merged with an array of miRNA-target predictions (comprising 12 distinct algorithms) obtained from the miRWalk 2.0 database [27]. Then, 3′ UTR sequences of the subset of transcripts were obtained from ENSEMBL. These sequences were subsequently screened for the presence of distinct miR-1287-5p seed match types (8mer, 7mer-A1, 7mer-m8, 6mer, and offset 6mer sites) [8].

### Luciferase reporter assay

To confirm the direct interaction of miR-1287-5p and the putative target PIK3CB, a 65 nt region of the predicted 3′ UTR binding site of PIK3CB was cloned into a luciferase containing pEZX-MT06 Vector (Genecopoeia, Rockville, MD, USA), either the wild-type miR-1287-5p target sequence or the mutated sequence. An empty control plasmid (Genecopoeia) was used as a reference control. For the Luciferase assay, HEK cells were seeded in 24-well plates on the day prior to the transfection. After 24 h, cells were co-transfected with 200 ng pEZ-MT06miRNA reporter vector (wild-type = 5′ TGGGTGATCTCTCTGAGTCCTGGCAAC*ATCCAGCA*AAACTACTGCTTATTCTCCAAAGAATATTGG 3′ CS-HmiT011246-MT06–01; GeneCopoeia), mutated plasmid (5′ TGGGTGATCTCTCTGAGTCCTGGCAAC*ATTTATTA*AAACTACTGCTTATTCTCCAAAGAATATTGG 3′ CS-HmiT011246-MT06-01; GeneCopoeia) or negative control (empty plasmid CS-HmiT011246-MT06–01; GeneCopoeia), and 50 nM miR-1287-5p Mimic or AllStars Negative Control (Syn-hsa-miR-1287-5p miScript miRNA Mimic or All Stars Negative Control, Qiagen), using Lipofectamine 2000 Transfection Reagent (Thermo Scientific, Waltham, MA USA) and Opti-MEM Reduced Serum Medium (Thermo Scientific). Cells were harvested 24 h after transfection and the Luc-Pair Luciferase Assay Kit 2.0 was performed according to the user manual. Luminescence was measured using a luminometer (LUMIStar Omega, BMG LabTech, Ortenberg, Germany) in three independent biological replicates. The ratio of luminescence from the firefly luciferase to the *Renilla* luciferase was calculated, and the empty control plasmid was used to normalize the luciferase activity.

### PI3K inhibitor sensitivity assays

For selective PI3K inhibitor experiments, untreated or transiently transfected BC cells were additionally treated with 2 μM and 10 μM BYL719 (Alpelisib, a selective PI3Kα inhibitor) and 25 μM and 50 μM CAL-101 (Idelalisib, a selective p110δ inhibitor) in a 96-well plate. All inhibitors were purchased via Selleckchem.com (Eubio, Vienna, Austria). Exposure time was 96 h before applying the WST-1 assay and compared to untreated control cells.

### Statistics

All statistical analyses were performed using SPSS version 23 software (SPSS Inc., Chicago, IL, USA). Unpaired Student’s *t* test or Mann-Whitney *U* test was applied. A two-sided *p* < 0.05 was considered statistically significant.

## Results

**Fig. 1:**
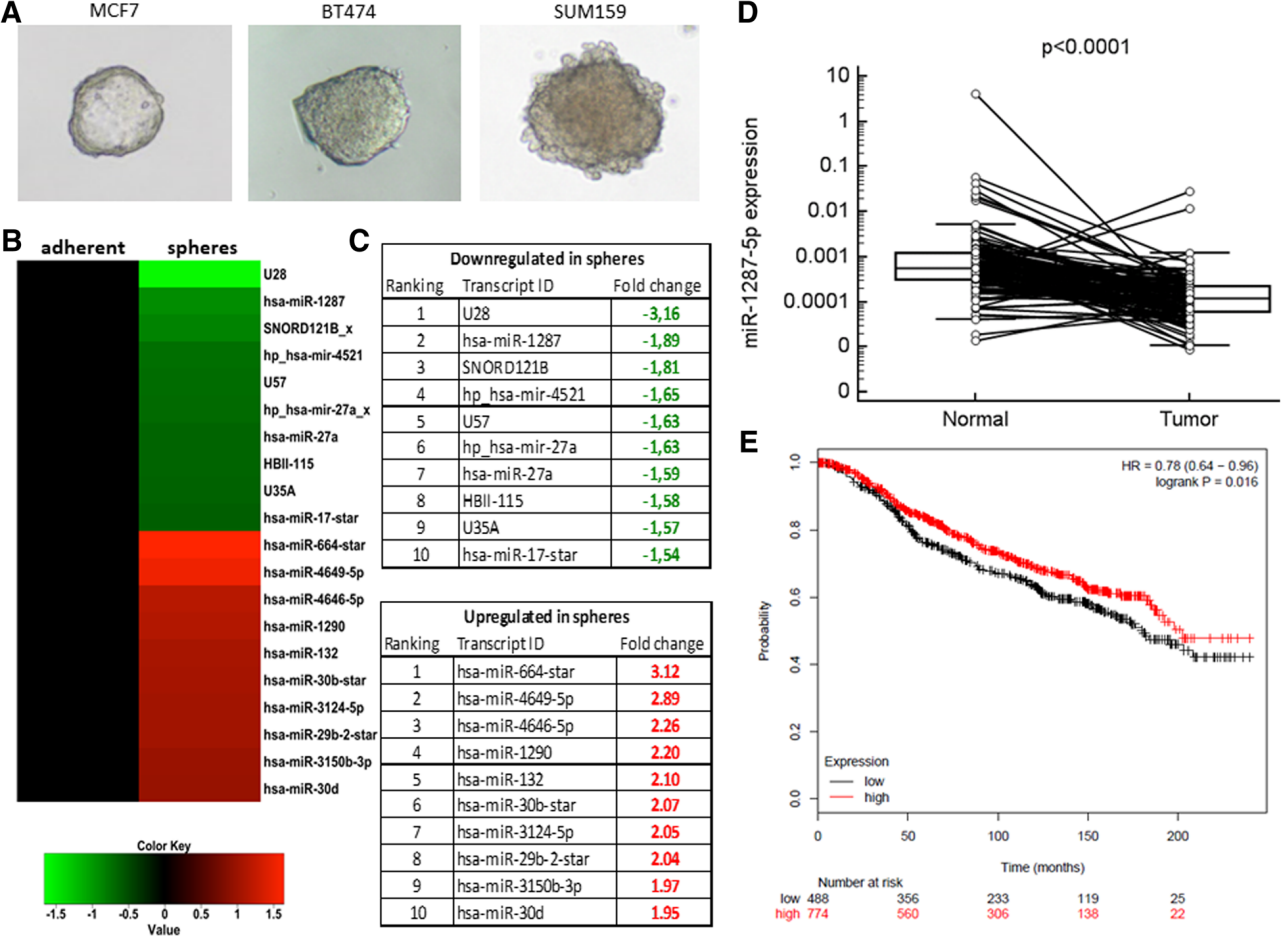
**a** Representative pictures of single mammospheres of the cell lines MCF7, BT474, and SUM159 used for profiling the whole miRNome. **b–c** Heat map and corresponding tables with fold changes of the top 10 down- and upregulated miRNAs in mammospheres compared to adherent growing cells. **d** A significantly lower expression level (4.6-fold downregulation) for miR-1287-5p was found in cancer tissue compared to corresponding normal tissue. **e** Low levels of miR-1287 were a negative prognostic factor for survival in an independent external large cohort of 1262 breast cancer patients (HR = hazard ratio)

In order to identify miRNAs with undetermined functions in breast carcinogenesis, we started to profile the whole miRNome for differences between mammospheres and parental adherent growing cells in cell lines representing different BC subtypes (Fig. 1a). Using this approach, we identified several differentially expressed miRNAs with currently unknown function in breast carcinogenesis (Fig. 1b, c). One of these miRNAs, miR-1287-5p, showed the highest level of downregulation in mammospheres and was therefore selected to be confirmed by independent qRT-PCR assay in four BC cell lines including two TNBC cell lines (Additional file 2: Figure S1A).

To translate the relevance of BC cell line findings to human cancer, we compared the expression levels of miR-1287-5p in a patient cohort of 131 matched cancer tissue and corresponding normal adjacent tissue samples. A significantly lower expression level (4.6 fold; *p* < 0.001, Wilcoxon test) of miR-1287-5p was detected in cancer tissue compared to normal breast tissue (Fig. 1d). This lower level of expression in cancer tissue was confirmed in a second independent cohort of 93 normal versus 916 cancer tissues obtained from TCGA dataset (*p* < 0.0001, Additional file 2: Figure S1B). Finally, we explored whether miR-1287-5p determines the fate of BC patients. Using a publicly available platform of 1262 patients [22], low levels of miR-1287-5p turned out to be a negative prognostic factor for survival in this large multi-data set cohort (*p* = 0.016, Fig. 1e). Three other miRNAs identified on our array analysis in the mammospheres (miR-27a-5p, miR-4521, and miR-3150) were also confirmed as significantly up- or downregulated in human BC samples of the TCGA dataset (Additional file 2: Figure S1C–E).

Based on our findings from clinical cohorts, we started a series of experiments to clarify the role of miR-1287-5p in BC biology. First of all, we measured miR-1287-5p in 11 different BC cell lines and confirmed the expression of this miRNA in all BC cells regardless of the underlying molecular subtype (Additional file 2: Figure S2A).

**Fig. 2:**
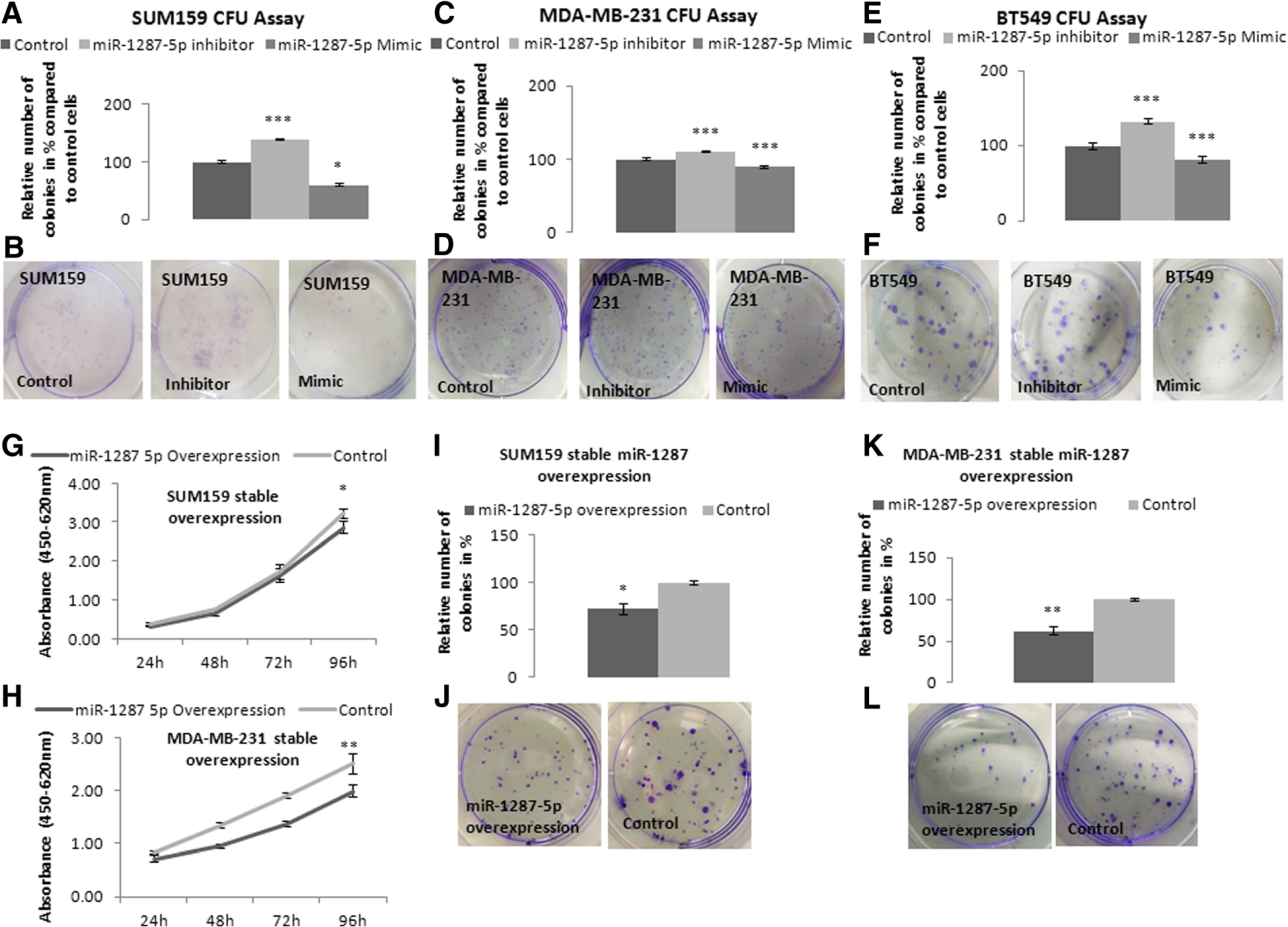
**a–f** Effects of transient overexpression or inhibition of miR-1287-5p on cellular growth using colony formation unit (CFU) assay in three different triple negative cell lines. Bar chart graphs represent relative number of colonies in percentage compared to control transfected cells (*n* = 3) (**a**, **c,** and **e**), representative pictures are shown (**b**, **d,** and **f**). Mir-1287-5p mimics led to a significant decrease in cellular growth, while miR-1287-5p inhibitor exerted the opposite effect. **g–h** Effect of stable overexpression of miR-1287-5p on cellular growth measured by WST-1 proliferation assay and (**i–l**) CFU assay in two different triple negative breast cancer cell lines. Stable overexpression of miR-1287-5p led to a significantly reduced cellular growth in the cell lines SUM159 and MDA-MB-231. *p < 0.05, **p < 0.01, ***p < 0.001

As BC is a very heterogeneous disease in terms of underlying biology, prognosis, and treatment strategy, we focused our study at that point on triple negative BC (TNBC). After successfully establishing transient gain (using a miR-1287-5p mimic; Additional file 2: Figure S3A) and loss (using a miR-1287-5p inhibitor; Additional file 2: Figure S3B) of function systems, we explored the effect on cellular growth in four independent TNBC cell lines (SUM159, MDA-MB-231, MDA-MB-468, and BT549). Ectopic overexpression of miR-1287-5p led to a significantly lower growth rate compared to control cells after 72 to 96 h (Additional file 2: Figure S4A–D). To confirm the results of this short-term cellular growth assay by a second independent assay, we used a colony formation unit (CFU) assay for cell lines SUM159, MDA-MB-231, and BT549 (MDA-MB-468 cells did not form any colonies under the experimental conditions used). This independent growth assay confirmed that transient overexpression of miR-1287-5p led to decreased number of colonies in all three cell lines (Fig. 2a–f). Moreover, using a miR-1287-5p inhibitor, we obtained the opposite effect and observed larger and higher numbers of colonies compared to the reference control (Fig. 2a–f). In addition, these growth inhibitory effects were not limited to TNBC cell lines as we could find similar effects in luminal A (MCF7) and HER2-positive (SKBR3) cells (Additional file 2: Figure S5A, B).

To further confirm the results of the transient transfection experiments in an additional model system, we generated SUM159 and MDA-MB-231 cell lines stably overexpressing the mature -5p form of miR-1287-5p (Additional file 2: Figure S5C, D). Stable overexpression of miR-1287-5p resulted in a significantly decreased cellular growth rate compared to control cells, in the WST-1 assay (Fig. 2g, h) and the CFU assay (Fig. 2i–l).

**Fig. 3:**
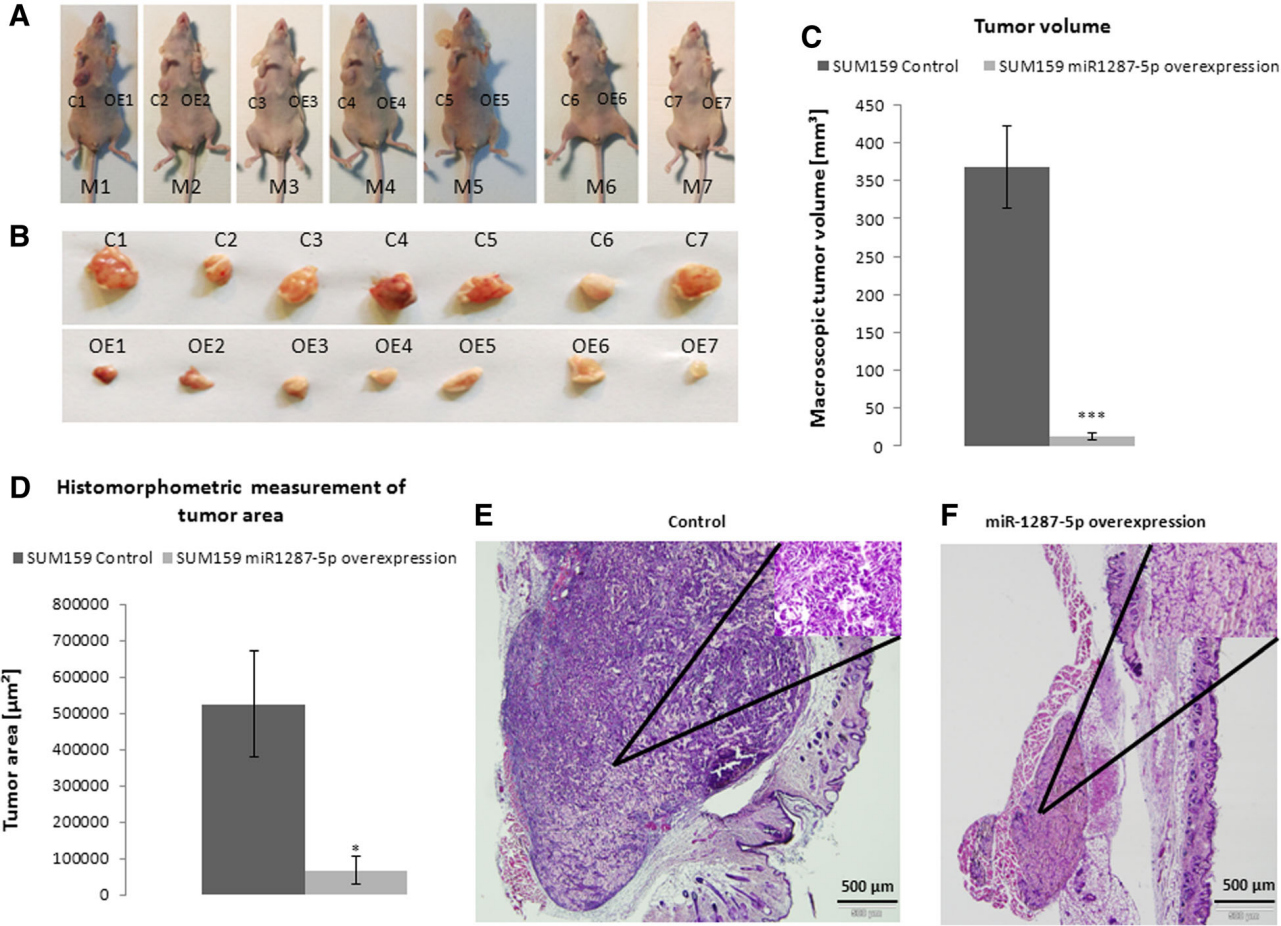
In vivo xenograft experiments of stable mature form of miR-1287-5p overexpressing SUM159 cells. MiR-1287-5p overexpressing cells were injected in the left mammary fat pad of nude mice compared to control SUM159 cells which were injected into the right site. **a–c** Cells with miR-1287-5p overexpression developed significantly smaller tumors compared to control cells. (**d**) Bar chart of histomorphometric measurements of the largest tumor area detected in HE-staining revealed significantly smaller tumor area in SUM159 miR-1287-5p overexpressing cells *p < 0.05, ***p < 0.001. (**e**-**f**) Representative histological pictures of control cells and miR-1287-5p overexpressing SUM159 (HE staining, × 4 magnification, inserts with x40 magnification)

For confirmation of the potentially anti-proliferative phenotype in vivo, we used SUM159 cells with stable overexpression of mature miR-1287-5p to evaluate tumor growth in the mammary fat pad of female nude mice. Macroscopic tumor assessment showed significantly smaller tumors in miR-1287-5p overexpressing cells compared to control cells (Fig. 3a–c). Histomorphometric analysis confirmed that the cross-sectional tumor area assessed at the maximum tumor diameter was significantly reduced in miR-1287-5p overexpressing BC cells (Fig. 3d, e). Hence, the in vivo experiment fully supports our in vitro findings that BC cells with high miR-1287-5p expression display significant reduction in tumor growth compared to cells with unchanged levels of miR-1287-5p expression.

We went on to investigate whether the precursor miR-1287 pheno-copies the same effect as the mature form of miR-1287-5p as in general mature -5p and -3p miRNAs are endogenously generated from their corresponding precursor-miRNAs. The mature -5p and -3p forms can have similar or opposing biological effects, and one of these miRNAs can actually dominate the biological effects of the precursor miRNA. For this purpose, we generated two different cell lines (SUM159 and MDA-MB-231) with miR-1287 precursor stable overexpression (Additional file 2: Figure S5E, F). WST-1 assay, CFU assay, and in vivo tumor growth demonstrated significantly decreased cellular growth after miR-1287 precursor overexpression for both tested BC cell lines (Additional file 2: Figure S6A–H) indicating concordant biological functions of precursor and mature miR-1287-5p.

After consistently demonstrating in multiple experimental and cellular model systems that miR-1287-5p expression regulates cellular growth in BC cells, we tested if the observed effects are regulated by apoptosis. Additional file 2: Figure S7 indicates that no signs for alterations in apoptosis (i.e., measured by effector caspase3/7 activity) were detected after miR-1287-5p expression changes. Caspase 3/7 activity was neither significantly altered in cells transiently transfected with miR-1287-5p mimic or inhibitor (Additional file 2: Figure S7A) nor in stable miR-1287 precursor (Additional file 2: Figure S7B) and stable mature form miR-1287-5p overexpressing cells (Additional file 2: Figure S7C) compared to control cells.

Cell cycle analysis was applied to further evaluate the cellular mode of action of miR-1287-5p in BC cells. Stable overexpression of miR-1287-5p led to an increased number of cells in G1-phase and decreased number of cells in the S phase (Additional file 2: Figure S8A, B). This phenotype was also observed in BC cell lines after forced transient miR-1287-5p overexpression, whereas the miR-1287-5p inhibitor led to the opposite phenotype (Additional file 2: Figure S8C, D).

Anchorage-independent growth (soft agar) assay, a commonly used in vitro feature for stemness of cells, was applied and showed that BC cells (SUM159 and MDA-MB-231) transiently transfected with miR-1287-5p mimic generated lower numbers of soft agar colonies, whereas cells treated with miR-1287-5p inhibitor showed the opposite phenotype (Additional file 2: Figure S9A, B). Again, the mature form of miR-1287-5p stably overexpressing cells generated significantly lower numbers of colonies in soft agar compared to control cells (Additional file 2: Figure S9C, D).

**Fig. 4:**
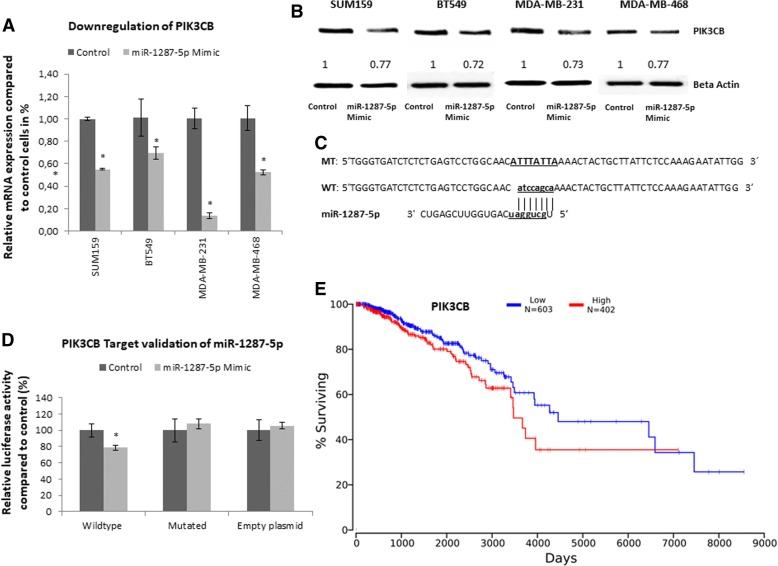
Target identification of miR-1287-5p in triple negative breast cancer cells. **a** qRT-PCR confirmed a significant downregulation of the *PIK3CB* mRNA in all four tested triple negative BC cell lines after forced ectopic miR-1287-5p overexpression after 48 h of transfection. **b** Western blot analysis confirmed a significant downregulation of PIK3CB on protein level after transient transfection of miR-1287-5p in all tested cell lines (SUM159, BT549, MDA-MB-231, and MDA-MB-468) after 48 h of transfection. Relative quantification (numbers above the lanes) of protein lanes was performed using ImageJ. **c** Predicted miR-1287-5p interaction site within 3′ untranslated region of *PIK3CB* mRNA. Two PIK3CB constructs were generated as indicated (WT = miR-1287 wild-type interacting site and MT = mutated interacting site). **d** Luciferase activity after co-transfection of the PIK3CB wild-type or mutated constructs and control/miR-1287-5p mimetic in HEK cells. Three independent biological experiments were performed, and the means and standard deviations are shown. **e** High-PIK3CB expression is associated with poor clinical outcome in 1005 BC patients of a TCGA dataset.*p < 0.05

After establishing this phenotype, we focused on the molecular mode of action and characterized possible interaction partners of miR-1287-5p. A microarray-based whole transcriptome analysis comparing SUM159 miR-1287-5p stable overexpressing cells against control cells was performed. As previously described for other miRNAs [28], a large number of mRNAs bearing at least one miR-1287-5p seed match in their 3′ UTR was repressed by miR-1287-5p overexpression (Additional file 2: Figure S9E). Based on these profiling results, we further focused on the most downregulated transcripts as these could be crucial direct interaction partners. This strategy brought us as many as 126 downregulated genes in miR-1287-5p overexpressing cells (Additional file 1: Table S1). Based on the in silico target prediction approach described in the “Materials and methods” section, we identified 78 putative interaction partners. We further selected six of them (*PIK3CB*, *LAYN*, *RAP2B*, *SMAD2*, *PLD5*, and *CORO2A* Additional file 3: Table S2), where literature-retrieved search implicated a general impact on tumor growth in any kind of cancer. Only three of the six array-based genes could be independently confirmed as significantly downregulated by independent qRT-PCR (*PIK3CB*, *LAYN*, and *RAP2B*; Additional file 2: Figure S9F). One promising candidate containing an 8mer seed match in in silico analysis was *PIK3CB*, which we found to be downregulated on mRNA and protein levels after transient overexpression of miR-1287-5p in all four TNBC cell lines (Fig. 4a, b). Bioinformatic prediction tools suggested a binding site of miR-1287-5p in the 3′ UTR of *PIK3CB* (Fig. 4c). To validate miR-1287-5p and *PIK3CB* interaction, a part of the 3′ UTR of *PIK3CB* predicted to interact with miR-1287-5p was cloned into a luciferase reporter vector and co-transfected with miR-1287-5p mimic into HEK cells. A significant reduction in the luciferase/*Renilla* ratio was observed for *PIK3CB* constructs transfected with synthetic miR-1287-5p but not with the scrambled RNA (Fig. 4d). Furthermore, the observed luciferase/*Renilla* reduction was abrogated when we co-transfected a luciferase reporter vector containing the mutated seed sequence of the 3′ UTR of *PIK3CB* with single exchanged nucleotides at the predicted site of interactions with miR-1287-5p (Fig. 4d). In order to prove the clinical relevance of PI3KCB in human BC, we performed a Kaplan-Meier curve analysis in 1005 BC patients of TCGA dataset. As is shown in Fig. 4e, a high PIK3CB expression is associated with poor clinical outcome (*p* = 0.0408). Using another publicly available dataset [23], we confirmed that high levels of PIK3CB are associated with poor recurrence-free survival (*n* = 3955, *p* < 0.001; Additional file 2: Figure S10) and poor overall survival (*n* = 1402, *p* = 0.029; Additional file 2: Figure S11).

**Fig. 5:**
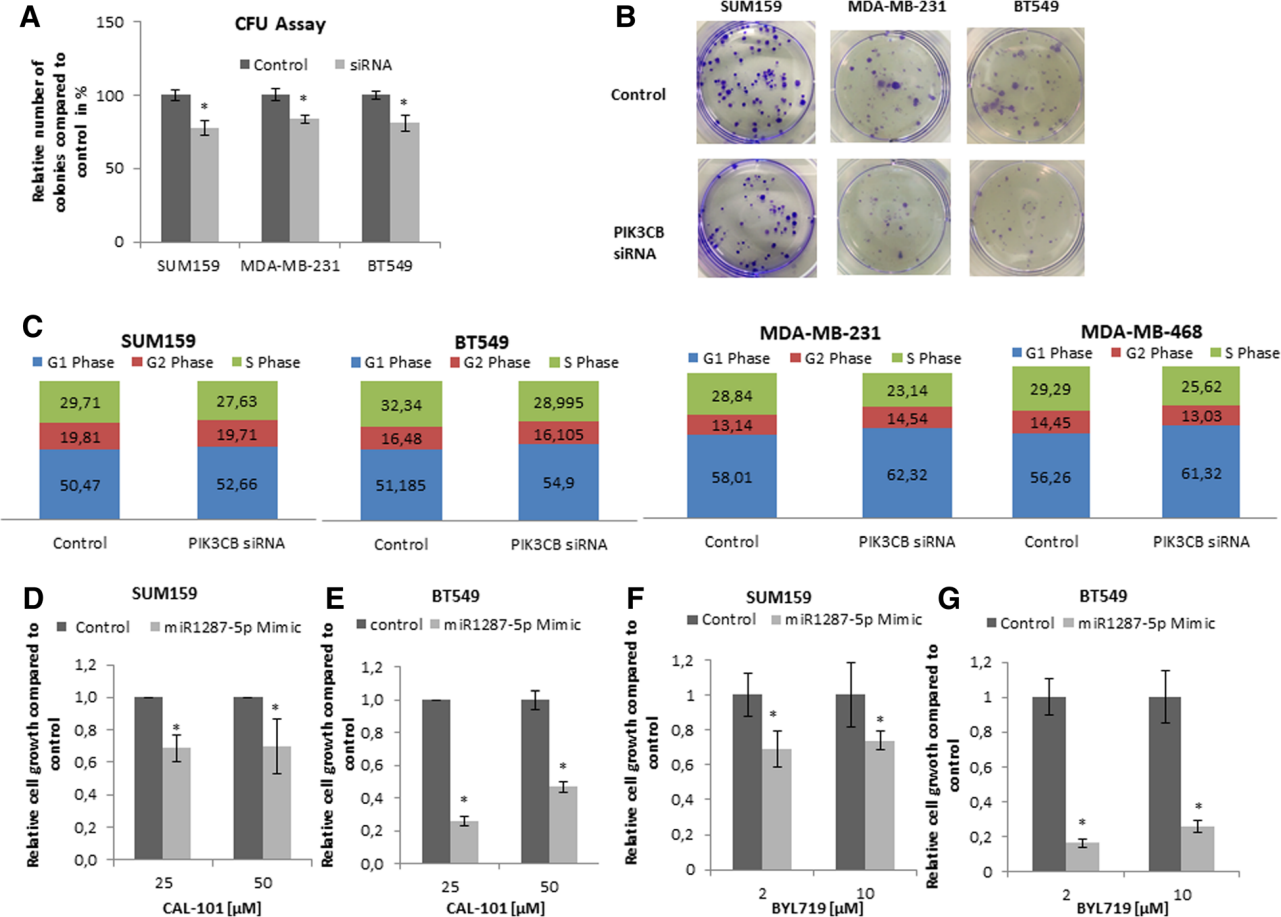
**a–c** Clonogenic assay of the cell lines SUM159, MDA-MB-231, and BT549 after transient silencing of the putative miR-1287-5p target *PIK3CB* leads to a similar phenotype compared to miR-1287 overexpression in the cell lines. Cells develop less colonies after PIK3CB silencing (**a**, **b**) and PIK3CB silencing also leads to a G1 Phase Arrest (**c**) in all four cell lines. **d–g** SUM159 and BT549 cells treated with two different concentrations of PI3Kinase inhibitors in combination with control scrambled RNA (10 μM of Allstar negative control) or the miR-1287-5p mimics (10 μM of miR-1287-5p mimics) (**d**, **e**) CAL101 (Idelalisib) and **f**, **g** BYL719 (Alpelisib). Cells treated with miR-1287-5p mimic are more sensitive to CAL-101 and BYL719 treatment in both tested cell lines compared to cells treated with the scrambled control RNA. *p < 0.05

Finally, to test whether *PIK3CB* expression pheno-copies the cellular effects of miR-1287-5p, we conducted knock-down experiments of *PIK3CB* using short-interfering RNA. Successful knockdown of *PIK3CB* was achieved on mRNA (Additional file 2: Figure S10A) and protein level (Additional file 2: Figure S10B). The reduced levels of PIK3CB lead to decreased cellular growth (Fig. 5a, b) and cell cycle shift from S phase towards the G1 phase (Fig. 5c).

To investigate potentially novel therapeutic vulnerabilities of TNBC cells based on our novel findings, we tested an approach combining miR-1287-5p mimic together with selective isoform-specific PI3K–inhibitors. Firstly, we tested the dose-dependent growth inhibitory effects of three different PI3K inhibitors CAL-101 (Idelalisib, GS-1101; a selective p110δ inhibitor), BYL719 (Alpelisib; a selective PI3Kα inhibitor), and GSK2636771 (a PI3Kβ-selective inhibitor). For CAL-101 and BYL719, we observed a dose-dependent cytotoxicity in all four tested TNBC cell lines, whereas for GSK2636771 no significant growth inhibition was observed in two of them, and therefore, this inhibitor was not further followed up (Additional file 2: Figures S12C–H and S13A–D). Finally, we treated two TNBC cell lines with different concentrations of CAL101 and BYL719, each of them in combination with miR-1287-5p mimics. As shown in Fig. 5d–g, combined treatment leads to higher cytotoxicity and reduced cellular growth in TNBC cells in comparison with the chemical compound only (combined with the miRNA mimics control). Cells transfected with miR-1287-5p mimic were more sensitive to CAL-101 and BYL719 treatment compared to cells transfected with the mimics control (*p* < 0.05).

## Discussion

In our present study, we started with an unbiased approach to identify novel miRNAs that have not yet been described in BC initiation and progression. MiR-1287-5p, which has not been systematically characterized in BC, was further evaluated for its clinical role and the cellular/molecular mode of action.

Data about the role of miR-1287-5p in BC is scarce, but the existence and downregulation of this miRNA seems to be consistent with our data. A previous study identified miR-1287 downregulation in BC tissue by miRNASeq profiling [29]. Another more recently published study similarly showed that miR-1287 was consistently downregulated in BC tissue as well as decreased in serum samples from BC patients [30].

The biological role of miR-1287 in BC is unknown. In retinoblastoma, miR-1287 was found to be downregulated [31], whereas Wang et al. reported that miRNA-1287 expression was increased in most cases of follicular lymphoma [32]. In larynx carcinoma, miR-1287 has been suggested as a potential biomarker for early diagnosis as it was found to be downregulated in larynx carcinoma samples compared to normal samples [33]. In cervical cancer, miR-1287 seems to be inactivated by DNA hypermethylation [34]. All these studies support the previous notion that biological functions of miRNAs are varying as one particular miRNA may act as an oncoMiR as well as a tumor suppressive miRNA, depending on the cellular and molecular context [14].

In line with the data previous reported for BC [29, 30], we confirmed the downregulation of miR-1287-5p in two independent external cohorts. Experimental data from multiple cellular and experimental models identified a growth inhibitory biological effect of miR-1287-5p in BC. Moreover, using precursor overexpressing experiments, we confirmed that the observed effects of the -5p mature form is the pre-dominant biological role. As shown by Almeida et al., the -5p and -3p mature form can show opposite and varying function and may generate different cellular phenotypes, an important aspect frequently omitted in numerous miRNA studies [35]. Though the growth inhibitory effects in some of our in vitro assays (e.g., WST-1) were rather slightly, the effects were consistent in multiple model systems and cell lines and the inhibition of tumor formation in vivo was biological meaningful. Of note, expression levels after transient transfection/stable transfection did not perfectly correlate with the strengthen of the observed phenotype, which may be explained by varying levels over time in transient transfection, varying transfection efficacy between experiments, and differences in target mRNA expression levels between different cell lines. Even more complex, endogenous competing long non-coding RNAs (ceRNAs) may vary between cell lines, and therefore may influence the non-linear observation between expression levels of miR-1287-5p after transfection and the observed phenotype changes.

Once discovering this consistent phenotype after miR-1287-5p and miR-1287 precursor overexpression, we sought to identify a molecular link between cellular growth and miR-1287-5p expression. Based on the assumption that our miRNA directly targets and thereby downregulates our gene of interest, we were able to identify several potential interaction partners of miR-1287-5p. In silico target prediction tools together with a comprehensive literature search of genes known to regulate cell growth narrowed the list down to one promising candidate gene, *PIK3CB*. It is a member of the PI3K (phosphoinositide 3-kinase)-pathway, and hyper- activation of this pathway contributes to human cancers progression [36]. Initiation of this signaling cascade induces cellular proliferation, motility, and survival in cancer cells [37]. Class IA PI3Ks are heterodimers consisting of a p85 regulatory subunit and a p110 catalytic subunit, and mammals display numerous isoforms of each subunit. The p110 catalytic subunits are p110α, β, or δ. *PIK3CB* (PI 3-kinase p110 beta/β) is one of the class IA PI3K isoforms of the catalytic subunit [38]. *PTEN* (phosphatase and tensin homolog deleted on chromosome 10) is one of the most frequently mutated tumor suppressor genes in human cancer and antagonizes the PI3K signaling pathway [39]. Downregulation of the *PIK3CB* gene in PTEN deficient cell lines resulted in PI3K-pathway inactivation and subsequent inhibition of growth in vitro and in vivo settings and this concludes that PTEN deficient tumors depend on PIK3CB [40]. In contrast to *PIK3CA*, where most activating point mutations occur [41] little is known about *PIK3CB*. Recent preclinical studies have shown that different PI3K isoforms play divergent roles in cellular signaling and cancer, and that is why isoform-selective inhibitors are now emerging as intensively explored agents in clinical trials [41, 42]. In a recently published study, the authors achieved PI3Kβ depletion by intra-tumoral injection of *PIK3CB* siRNA, which induced apoptosis and triggered regression of PTEN-mutant tumors even more efficiently than PI3Kβ inhibition in urothelial bladder carcinoma [43].

Finally, we approached the field of selective PI3K inhibitors together with miR-1287-5p mimics to tackle TNBC cells. Targeting the PI3K/AKT pathway in TNBC is an ongoing and rapidly expanding area of knowledge [42]. We observed an augmentation of growth inhibition by combining miR-1287-5p with the selective inhibitors idelalisib and alpelisib. Both agents are either currently approved for non-Hodkin lymphoma (idelalisib) [44] or tested in BC (alpelisib) [45]. Though the observed effects are interesting, this data should be interpreted with caution. Further preclinical testing including other BC subtypes (especially the estrogen positive one) and in vivo studies are necessary to further follow this concept.

## Conclusions

In summary, we substantiated previous reports about a loss of function of miR-1287 in breast cancer. Furthermore, we identified the cellular and biological role of miR-1287-5p and identified potential molecular interaction partners. A novel role for PI3KCB in breast cancer and potential druggable combination approaches warrants to further study the role of miR-1287-5p as a possible therapeutic target in BC to develop improved therapeutic approaches for TNBC patients.

## Additional files

Additional file 1: **Table S1.** Primer sequences used for quantitative RT-PCR analysis. (DOCX 15 kb)

Additional file 2: **Figure S1.** (A) Measurement of miR-1287-5p in mammospheres compared to control cells by quantitative RT-PCR in four BC cell lines including two triple negative (SUM159 and MDA-MB-231) confirms the results of the microarray analysis. MiR-1287-5p is downregulated in mammospheres compared to adherent growing parental cells; *p < 0.05, **p < 0.01 . (B) Lower miR-1287-5p expression levels in cancer tissue compared to normal tissue could be confirmed in a second independent cohort using breast cancer patients from the TCGA dataset. (C–E) Other microRNAs, including miR-27, miR-3150, and miR-4521, are significantly up- or downregulated in normal breast versus cancer tissue in the TCGA dataset. Figure S2 (A) Relative miR-1287-5p expression levels in 11 different breast cancer cell lines ordered by the subtype; miR-1287-5p could be detected in all BC cell lines regardless of the molecular subtype (T-47D, MCF-7 and KPL-1 for luminal A; BT474 luminal B; HCC1937, SUM159, MDA-MB-231, MDA-MB-468, BT549 Triple negative; SKBR3 and HCC1419 HER2/neu expressing cell lines). Figure S3 Confirmation of expression changes of miR-1287-5p by quantitative RT-PCR in four different breast cancer cell lines (A) Transient overexpression of miR-1287-5p using a miR-1287-5p mimic and (B) transient silencing using miR-1287-5p inhibitor. *p < 0.05, **p < 0.01, ***p < 0.001. Figure S4 WST-1 assay in four different triple negative breast cancer cell lines after transient miR-1287-5p overexpression. Line graphs represent cell growth after transient transfection in the cell lines SUM159, BT549, MDA-MB-468, and MDA-MB-231. miR1287-5p overexpression led to significantly decreased cellular growth rates in all tested cell lines (A) SUM159 (*p* = 0.016825), (B) BT549 (*p* = 0.0001), (C) MDA-MB-468 (*p* = 0.019857), and (D) MDA-MB-231 (*p* = 0.020009). *p < 0.05, ***p < 0.001. Figure S5 (A, B) Cellular growth rate in non-triple negative breast cancer cell lines upon manipulation of miR-1287-5p expression level. miR-1287 mimic led to a reduced cellular growth rate, whereas the miR-1287-5p inhibitor generated more colonies in (A) luminal A MCF7 cells and (B) HER2-positive SKBR3 cells. (C) Quantitative RT-PCR data upon stable miR-1287-5p mature form overexpression in (C) SUM159 cells and (D) MDA-MB-231 cells. (E, F) Expression levels of miR-1287-5p upon stable miR-1287 precursor overexpression in (E) SUM159 and (F) MDA-MB-231 cells. *p < 0.05, **p < 0.01, ***p < 0.001. Figure S6 Biological effects of miR-1287 precursor overexpression using the WST-1 assay, Colony Formation Unit assay and in vivo Xenograft growth assay. (A–C) Overexpression of the precursor led to a significantly reduced cellular growth in the cell line SUM159 in vitro and (D,E) in vivo Xenograft formation. (F–H) Independent confirmation in the MDA-MB-231. ***p < 0.001. Figure S7 Caspase 3/7 assay was used to measure apoptotic activity in (A) transiently transfected cell lines SUM159, MDA-MB-231, MDA-MB-468, and BT549 and (B) stable miR-1287 precursor overexpressing cell lines SUM159 and MDA-MB-231 as well as (C) in miR-1287-5p overexpressing cells. Caspase 3/7 activity was normalized to control transfected cells. No significant differences in caspase activity could be detected, indicating that altered miR-1287-5p expression has no effects on apoptosis. Figure S8 Cell cycle of cells with overexpression or inhibition of miR-1287-5p expression. (A–B) Stable overexpression of miR-1287-5p resulted in significantly increased cells in G1-phase compared to control cells in the cell line (A) SUM159 and (B) MDA-MB-231. (C–D) Transient overexpression and inhibition of miR-1287-5p resulted in increased number of cells in G1-phase for the mimic and decreased number of cells in G1-phase for the inhibitor compared to control cells, respectively. Figure S9 (A,B). Transiently forced expression of miR-1287-5p in two breast cancer cell lines led to significantly less number of colonies in the soft agar, whereas (C, D) transient silencing by an inhibitor induced more and bigger colonies in soft agar. (E) Cumulative distributions of mRNA fold changes between miR-1287-5p overexpressing and control SUM159 cells. The distributions of transcripts without (black, *n* = 12,210) or with (red, *n* = 236) at least one miR-1287-5p seed match (type 8mer) in their 3′ UTR were significantly different (*p* = 7E-5) as assessed by a one-sided Kolmogorov-Smirnov test. (F) qRT-PCR validation of three putative miR-1287-5p interacting candidate genes PIK3CB, LAYN and RAP2B in the cell line SUM159. *p < 0.05, **p < 0.01. Figure S10 High PI3KCB expression levels are associated with poor recurrence-free survival in patients with breast cancer (*n* = 3955). Figure S11 High PI3KCB expression levels are associated with poor overall survival in patients with breast cancer (*n* = 1402). Figure S12 (A) qRT-PCR validation of three putative miR-1287-5p interacting candidate genes *PIK3CB*, *LAYN* and *RAP2B* in the cell line SUM159. Successful knock-down by short interfering RNA against PIK3CB was demonstrated by (B) qRT-PCR and (C) Western Blot analysis upon transient transfection. A significantly decreased PIK3CB level could be achieved compared to control transfected cells. (D–I): Growth inhibitory effects of dose-depending exposure of selective PI3K-inhibitors on the cell lines SUM159 and MDA-MB-231. (D–E) CAL-101, (F–G) BYL719, and (H–I) GSK2636771 ***p < 0.001. Figure S13 Growth inhibitory effects of dose-depending exposure of selective PI3K-inhibitors on the cell lines BT549 and MDA-MB-468. (A–B) CAL-101, (C–D) BYL719 (PDF 712 kb)

Additional file 3: **Table S2.** Downregulated mRNAs after miR-1287 overexpression in the cell line SUM159. This list shows 126 mRNAs that were at least 1.5-fold downregulated with a *p*-value of< 0.05. (DOCX 18 kb)

## Abbreviations

AKT: Protein kinase B
BC: Breast cancer
BRCA1: Breast cancer gene 1
CFU: Colony formation unit
CORO2A: Coronin 2A
CSC: Cancer stem cell
EGF: Epidermal growth factor
ER: Estrogen receptor
FBS: Fetal bovine serum
FGF: Fibroblast growth factor
GAPDH: Glyceraldehyde-3-phosphate dehydrogenase
LAYN: Layilin
miRNA: MicroRNA
PARP: Poly (ADP-ribose)
PI3KCA/B: Phosphatidylinositol 4,5-bisphosphate 3-kinase catalytic subunit alpha/beta isoform
PLD5: Inactive phospholipase D5
PR: Progesterone receptor polymerase
PTEN: Phosphatase and tensin homolog deleted on chromosome 10
RAP2B: Ras-related protein Rap-2b
RIPA: Radioimmunoprecipitation assay
SMAD2: Mothers against decapentaplegic homolog 2
TCGA: The Cancer Genome Atlas
TNBC: Triple negative breast cancer
U6: U6 protein
WST-1: Cell proliferation assay
