# Supplementary material for: MiR-1287-5p inhibits triple negative breast cancer growth by interaction with phosphoinositide 3-kinase CB, thereby sensitizing cells for PI3Kinase inhibitors
Source: Breast Cancer Res. 2019 Feb 1;21:20. doi: 10.1186/s13058-019-1104-5 (PMC6359814; doi:10.1186/s13058-019-1104-5)
Supplement: Supplementary file 1 — Table S1. Primer sequences used for quantitative RT-PCR analysis. (DOCX 15 kb) [file 13058_2019_1104_MOESM1_ESM.docx]

Table S1: Primer sequences for qRT-PCR analysis.

| **Primer ID** | **Sequence 5'- 3'** |
| --- | --- |
| **GAPDH_fw** | AAG GTC GGA GTC AAC GGA TTT |
| **GAPDH_rev** | ACC AGA GTT AAA AGC AGC CCT G |
| **U6_fw** | CTC GCT TCG GCA GCA CA |
| **U6_rev** | AAC GCT TCA CGA ATT TGC GT |
| **PIK3CB_fw** | AGA TCG CTC TGG CCT CAT TG |
| **PIK3CB_rev** | TCC AGG TCA TCC CCA GAG TT |
| **SMAD2_fw** | GTG CCT CGC GCC CTA A |
| **RAP2B_fw** | AAG CCT CGG TAG ACG AGC TA |
| **RAP2B_rev** | GTC GGA TGC GTT TGG CTT TT |
| **PLD5_fw** | GGG AAG GAA GAT TGG TCG CA |
| **PLD5_rev** | CCA CCA GGG CAA TTC GAC A |
| **LAYN_fw** | CGG GAA CCG CGC TAC AG |
| **LAYN_rev** | GAG GTC CAA ATC CGA GGC ACT |
| **CORO2A_fw** | GCC CAG CCT GGA CAA ACA AT |
| **CORO2A_rev** | GGG TGC CAT GAC ATC TGC TT |
