# Supplementary material for: MiR-1287-5p inhibits triple negative breast cancer growth by interaction with phosphoinositide 3-kinase CB, thereby sensitizing cells for PI3Kinase inhibitors
Source: Breast Cancer Res. 2019 Feb 1;21:20. doi: 10.1186/s13058-019-1104-5 (PMC6359814; doi:10.1186/s13058-019-1104-5)
Supplement: Supplementary file 2 — Figure S1. (A) Measurement of miR-1287-5p in mammospheres compared to control cells by quantitative RT-PCR in four BC cell lines including two triple negative (SUM159 and MDA-MB-231) confirms the results of the microarray analysis. MiR-1287-5p is downregulated in mammospheres compared to adherent growing parental cells; *p < 0.05, **p < 0.01 . (B) Lower miR-1287-5p expression levels in cancer tissue compared to normal tissue could be confirmed in a second independent cohort using breast cancer patients from the TCGA dataset. (C–E) Other microRNAs, including miR-27, miR-3150, and miR-4521, are significantly up- or downregulated in normal breast versus cancer tissue in the TCGA dataset. Figure S2 (A) Relative miR-1287-5p expression levels in 11 different breast cancer cell lines ordered by the subtype; miR-1287-5p could be detected in all BC cell lines regardless of the molecular subtype (T-47D, MCF-7 and KPL-1 for luminal A; BT474 luminal B; HCC1937, SUM159, MDA-MB-231, MDA-MB-468, BT549 Triple negative; SKBR3 and HCC1419 HER2/neu expressing cell lines). Figure S3 Confirmation of expression changes of miR-1287-5p by quantitative RT-PCR in four different breast cancer cell lines (A) Transient overexpression of miR-1287-5p using a miR-1287-5p mimic and (B) transient silencing using miR-1287-5p inhibitor. *p < 0.05, **p < 0.01, ***p < 0.001. Figure S4 WST-1 assay in four different triple negative breast cancer cell lines after transient miR-1287-5p overexpression. Line graphs represent cell growth after transient transfection in the cell lines SUM159, BT549, MDA-MB-468, and MDA-MB-231. miR1287-5p overexpression led to significantly decreased cellular growth rates in all tested cell lines (A) SUM159 (p = 0.016825), (B) BT549 (p = 0.0001), (C) MDA-MB-468 (p = 0.019857), and (D) MDA-MB-231 (p = 0.020009). *p < 0.05, ***p < 0.001. Figure S5 (A, B) Cellular growth rate in non-triple negative breast cancer cell lines upon manipulation of miR-1287-5p expression level. miR-1287 [file 13058_2019_1104_MOESM2_ESM.pdf]

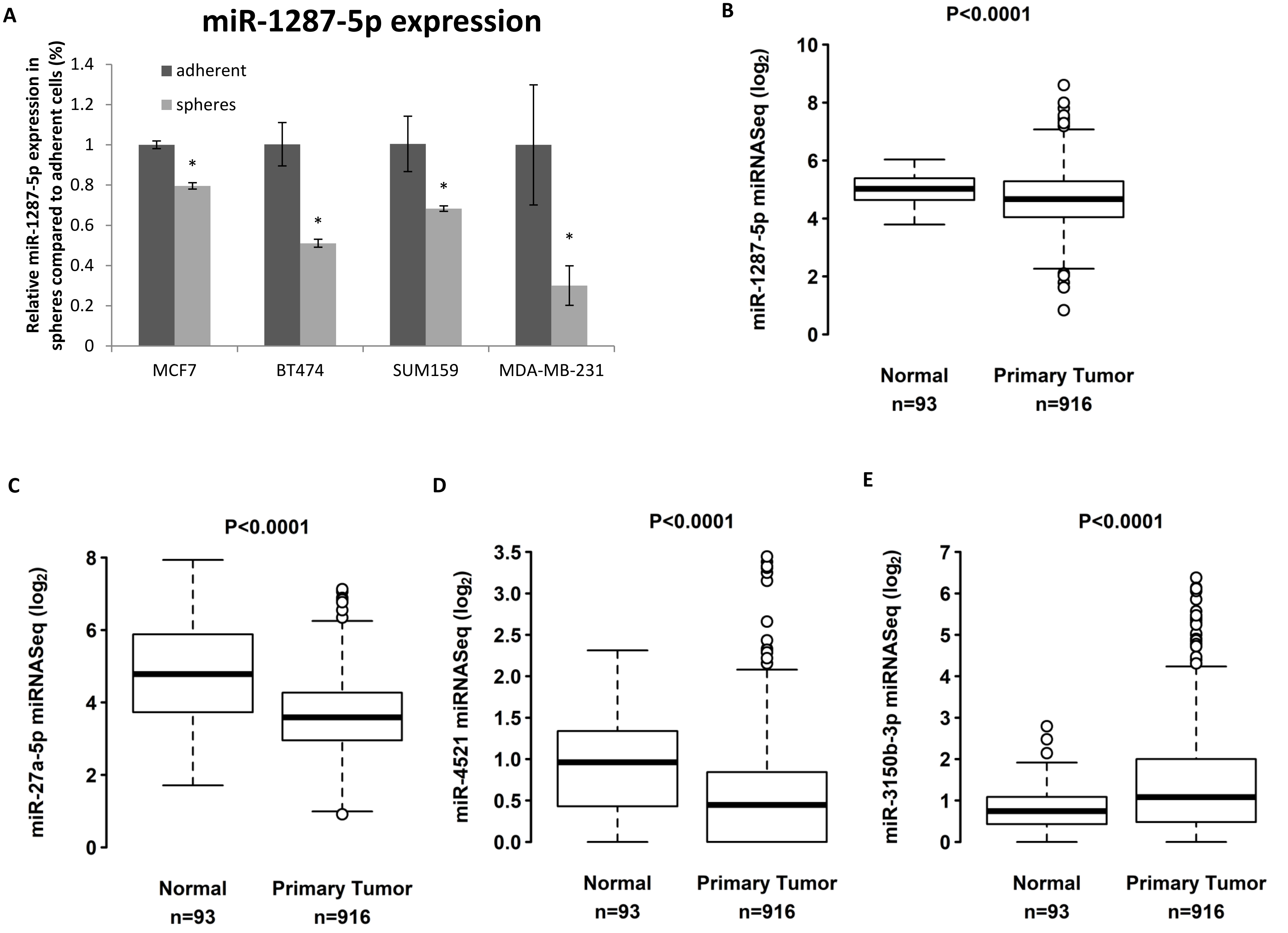
A
miR-1287-5p expression
Relative miR-1287-5p expression in spheres compared to adherent cells (%)
adherent
spheres
1.4
1.2
1
0.8
0.6
0.4
0.2
0
*
MCF7
BT474
SUM159
MDA-MB-231
B
P<0.0001
miR-1287-5p miRNASeq (log2)
10
8
6
4
2
0
Normal
n=93
Primary Tumor
n=916
C
P<0.0001
miR-27a-5p miRNASeq (log2)
Normal
n=93
Primary Tumor
n=916
D
P<0.0001
miR-4521 miRNASeq (log2)
3.5
3.0
2.5
2.0
1.5
1.0
0.5
0.0
Normal
n=93
Primary Tumor
n=916
E
P<0.0001
miR-3150b-3p miRNASeq (log2)
7
6
5
4
3
2
1
0
Normal
n=93
Primary Tumor
n=916

A

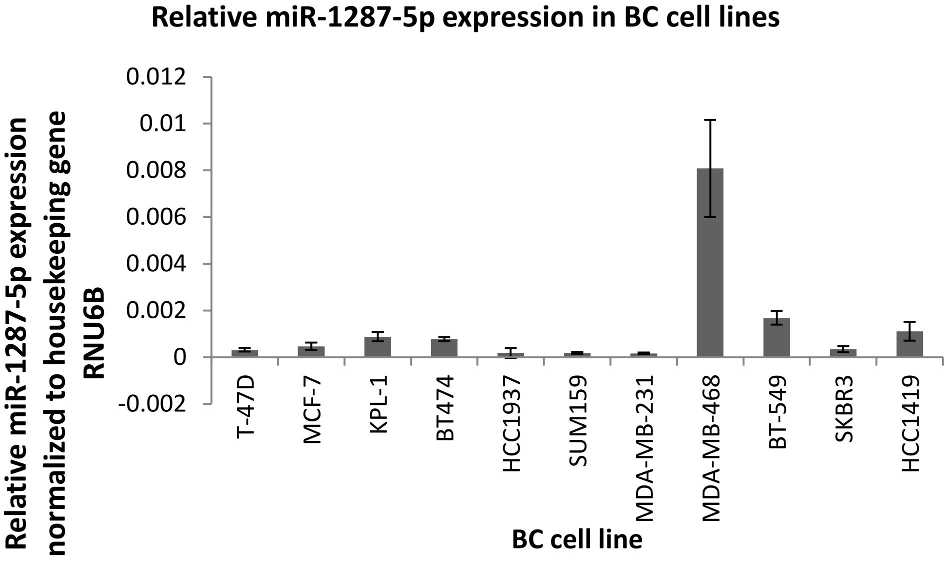
Relative miR-1287-5p expression in BC cell lines
Relative miR-1287-5p expression normalized to housekeeping gene RNU6B
0.012
0.01
0.008
0.006
0.004
0.002
0
-0.002
T-47D
MCF-7
KPL-1
BT474
HCC1937
SUM159
MDA-MB-231
MDA-MB-468
BT-549
SKBR3
HCC1419
BC cell line

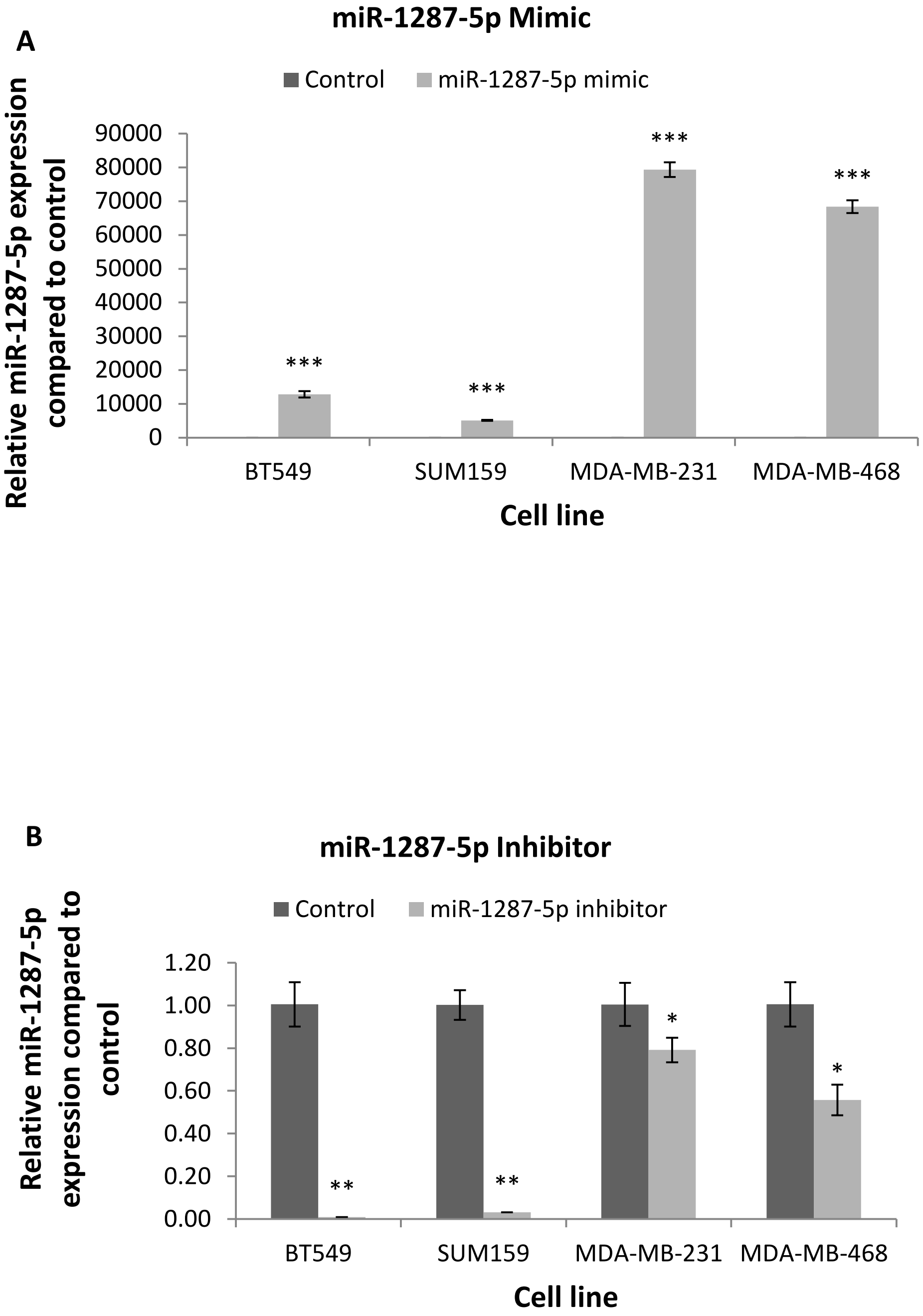

A
miR-1287-5p Mimic
Control
miR-1287-5p mimic
Relative miR-1287-5p expression compared to control
90000
80000
70000
60000
50000
40000
30000
20000
10000
0
***
***
***
***
BT549
SUM159
MDA-MB-231
MDA-MB-468
Cell line
B
miR-1287-5p Inhibitor
Control
miR-1287-5p inhibitor
Relative miR-1287-5p expression compared to control
1.20
1.00
0.80
0.60
0.40
0.20
0.00
**
**
*
*
BT549
SUM159
MDA-MB-231
MDA-MB-468
Cell line

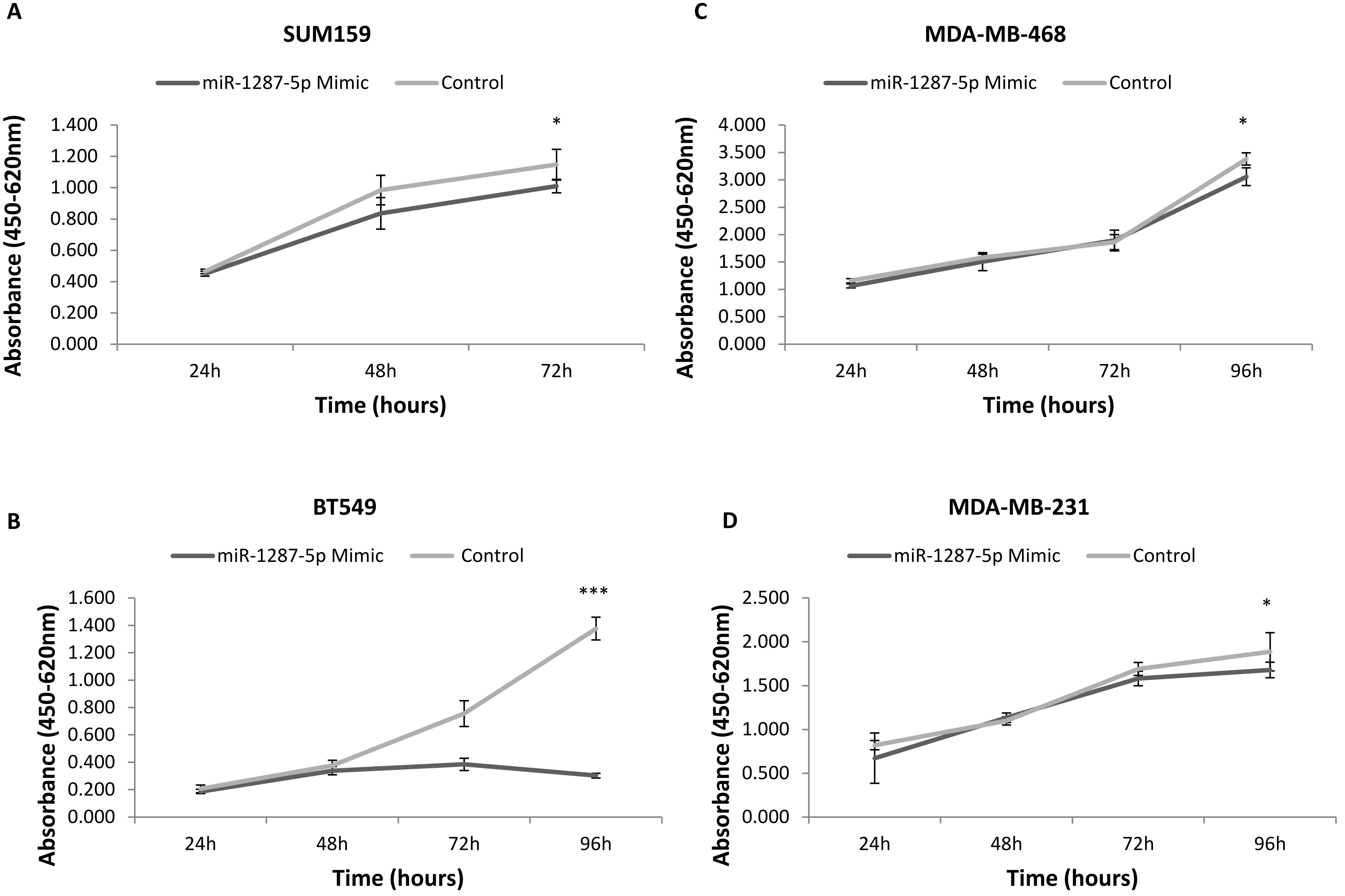
A
SUM159
miR-1287-5p Mimic
Control
Absorbance (450-620nm)
1.400
1.200
1.000
0.800
0.600
0.400
0.200
0.000
*
24h
48h
72h
Time (hours)
C
MDA-MB-468
miR-1287-5p Mimic
Control
Absorbance (450-620nm)
4.000
3.500
3.000
2.500
2.000
1.500
1.000
0.500
0.000
*
24h
48h
72h
96h
Time (hours)
B
BT549
miR-1287-5p Mimic
Control
Absorbance (450-620nm)
1.600
1.400
1.200
1.000
0.800
0.600
0.400
0.200
0.000
***
24h
48h
72h
96h
Time (hours)
D
MDA-MB-231
miR-1287-5p Mimic
Control
Absorbance (450-620nm)
2.500
2.000
1.500
1.000
0.500
0.000
*
24h
48h
72h
96h
Time (hours)

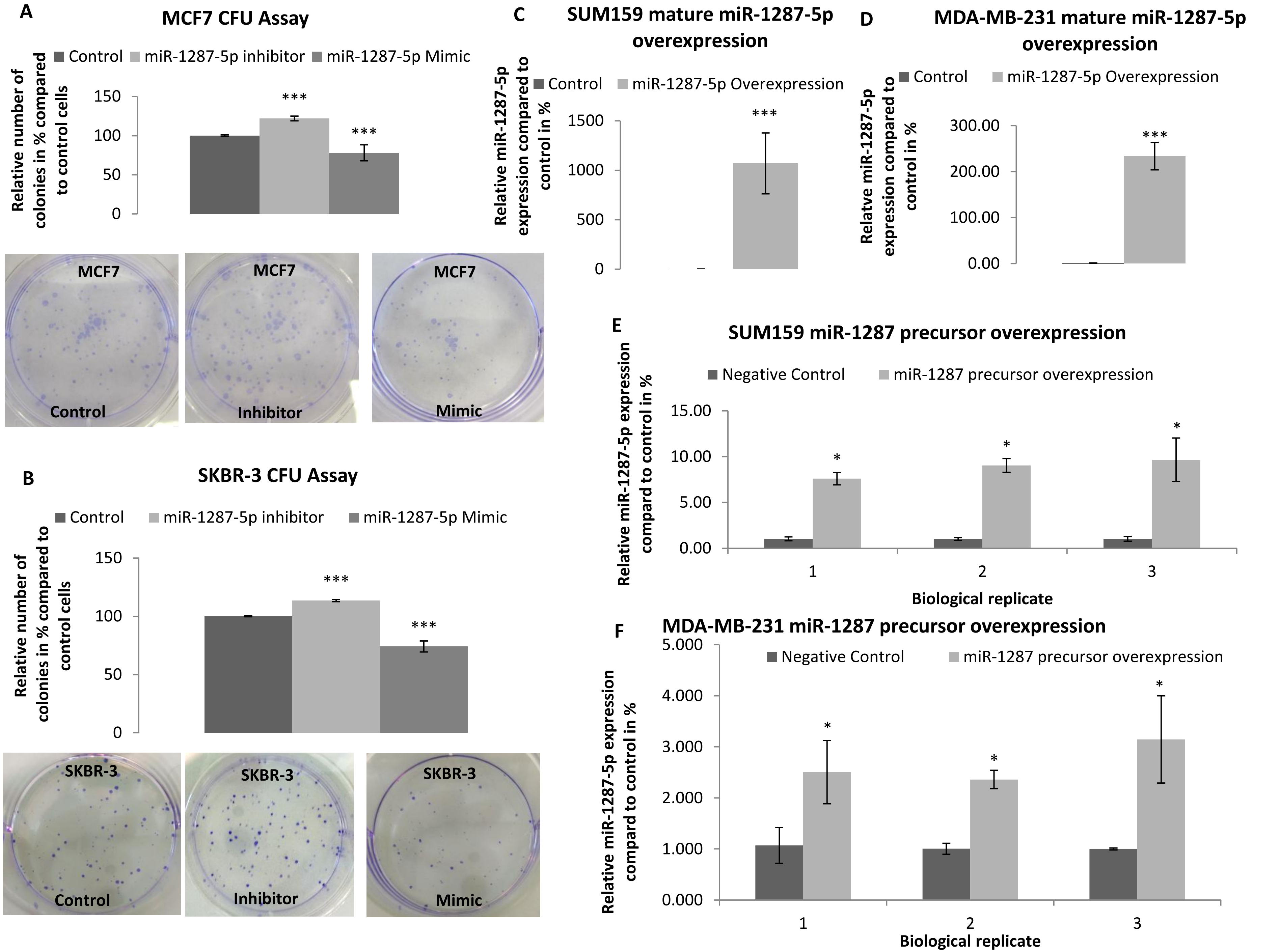
A
MCF7 CFU Assay
Control
miR-1287-5p inhibitor
miR-1287-5p Mimic
Relative number of colonies in % compared to control cells
150
100
50
0
***
***
MCF7
MCF7
MCF7
Control
Inhibitor
Mimic
B
SKBR-3 CFU Assay
Control
miR-1287-5p inhibitor
miR-1287-5p Mimic
Relative number of colonies in % compared to control cells
150
100
50
0
***
***
SKBR-3
SKBR-3
SKBR-3
Control
Inhibitor
Mimic
C
SUM159 mature miR-1287-5p overexpression
Control
miR-1287-5p Overexpression
Relative miR-1287-5p expression compared to control in %
1500
1000
500
0
***
D
MDA-MB-231 mature miR-1287-5p overexpression
Control
miR-1287-5p Overexpression
Relatve miR-1287-5p expression compared to control in %
300.00
200.00
100.00
0.00
***
E
SUM159 miR-1287 precursor overexpression
Negative Control
miR-1287 precursor overexpression
Relative miR-1287-5p expression compard to control in %
15.00
10.00
5.00
0.00
*
*
*
1
2
3
Biological replicate
F
MDA-MB-231 miR-1287 precursor overexpression
Negative Control
miR-1287 precursor overexpression
Relative miR-1287-5p expression compard to control in %
5.000
4.000
3.000
2.000
1.000
0.000
*
*
*
1
2
3
Biological replicate

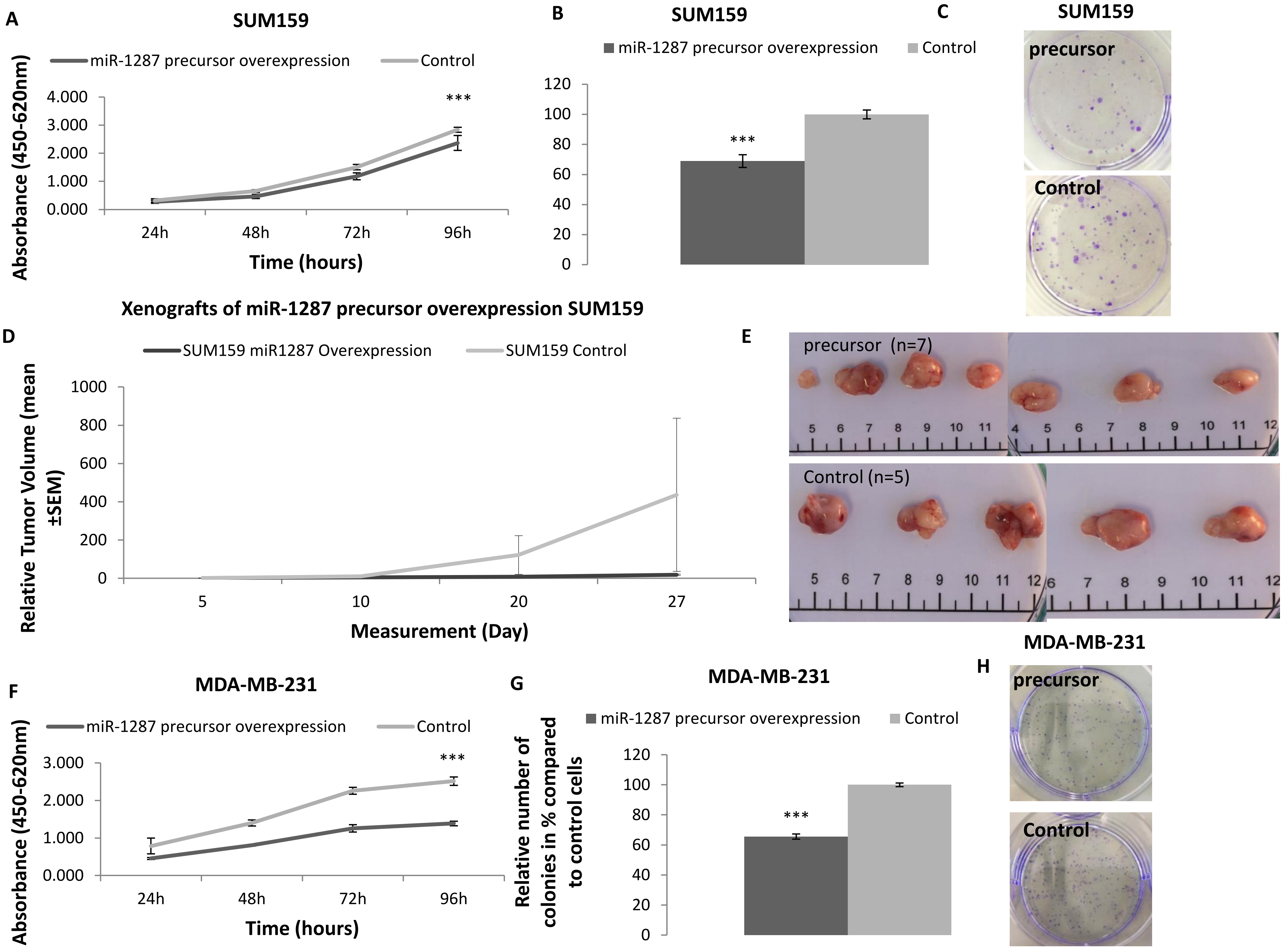

A
SUM159
miR-1287 precursor overexpression
Control
Absorbance (450-620nm)
4.000
3.000
2.000
1.000
0.000
***
24h
48h
72h
96h
Time (hours)
B
SUM159
miR-1287 precursor overexpression
Control
120
100
80
60
40
20
0
***
C
SUM159
precursor
Control
Xenografts of miR-1287 precursor overexpression SUM159
D
SUM159 miR1287 Overexpression
SUM159 Control
Relative Tumor Volume (mean ±SEM)
1000
800
600
400
200
0
5
10
20
27
Measurement (Day)
E
precursor (n=7)
Control (n=5)
F
MDA-MB-231
miR-1287 precursor overexpression
Control
Absorbance (450-620nm)
3.000
2.000
1.000
0.000
***
24h
48h
72h
96h
Time (hours)
G
MDA-MB-231
miR-1287 precursor overexpression
Control
Relative number of colonies in % compared to control cells
120
100
80
60
40
20
0
***
H
MDA-MB-231
precursor
Control

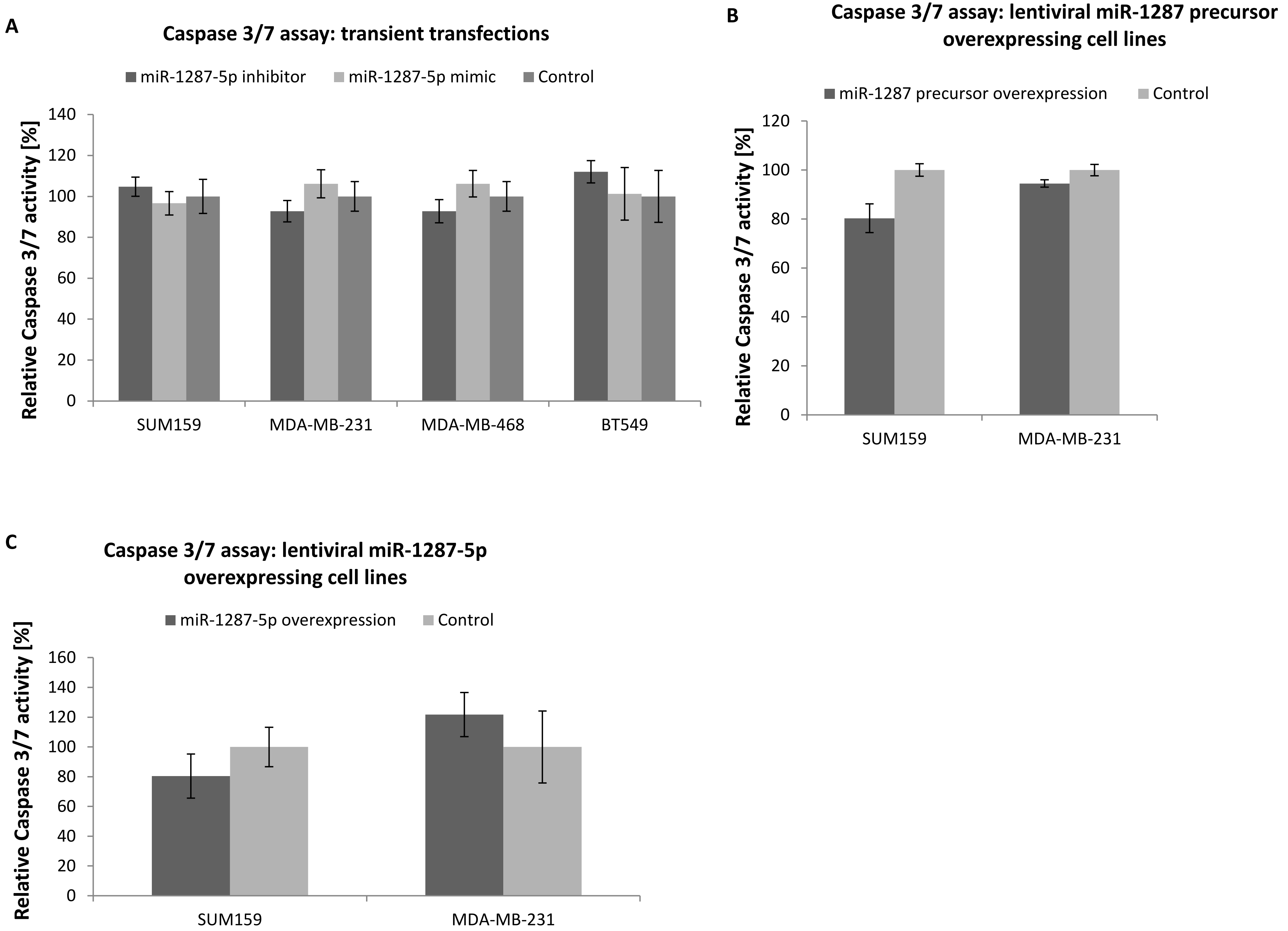

A
Caspase 3/7 assay: transient transfections
miR-1287-5p inhibitor
miR-1287-5p mimic
Control
Relative Caspase 3/7 activity [%]
0
20
40
60
80
100
120
140
SUM159
MDA-MB-231
MDA-MB-468
BT549
B
Caspase 3/7 assay: lentiviral miR-1287 precursor overexpressing cell lines
miR-1287 precursor overexpression
Control
Relative Caspase 3/7 activity [%]
0
20
40
60
80
100
120
SUM159
MDA-MB-231
C
Caspase 3/7 assay: lentiviral miR-1287-5p overexpressing cell lines
miR-1287-5p overexpression
Control
Relative Caspase 3/7 activity [%]
0
20
40
60
80
100
120
140
160
SUM159
MDA-MB-231

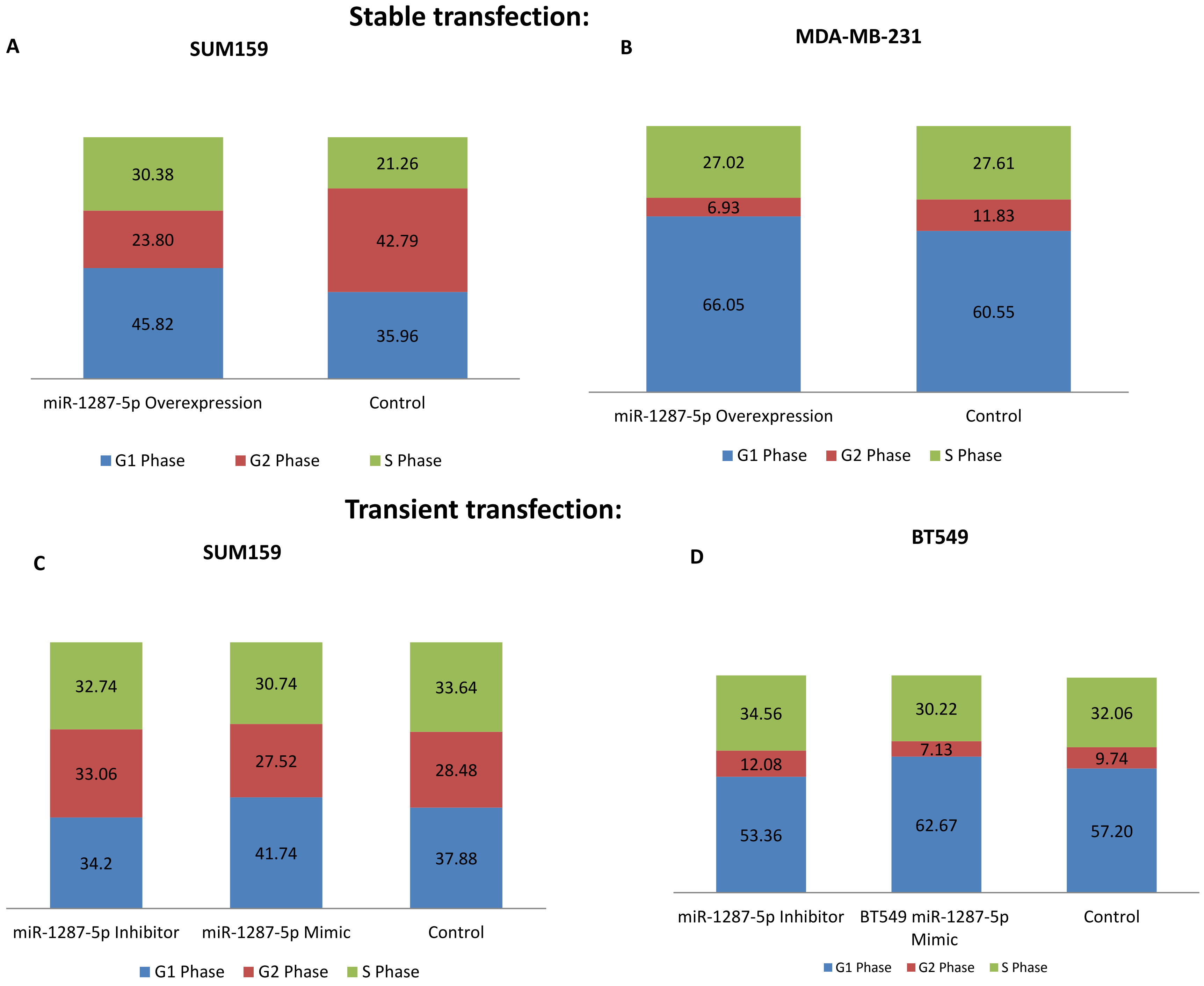
Stable transfection:
A
SUM159
30.38
23.80
45.82
21.26
42.79
35.96
miR-1287-5p Overexpression
Control
G1 Phase
G2 Phase
S Phase
B
MDA-MB-231
27.02
6.93
66.05
27.61
11.83
60.55
miR-1287-5p Overexpression
Control
G1 Phase
G2 Phase
S Phase
Transient transfection:
C
SUM159
32.74
33.06
34.2
30.74
27.52
41.74
33.64
28.48
37.88
miR-1287-5p Inhibitor
miR-1287-5p Mimic
Control
G1 Phase
G2 Phase
S Phase
D
BT549
34.56
12.08
53.36
30.22
7.13
62.67
32.06
9.74
57.20
miR-1287-5p Inhibitor
BT549 miR-1287-5p Mimic
Control
G1 Phase
G2 Phase
S Phase

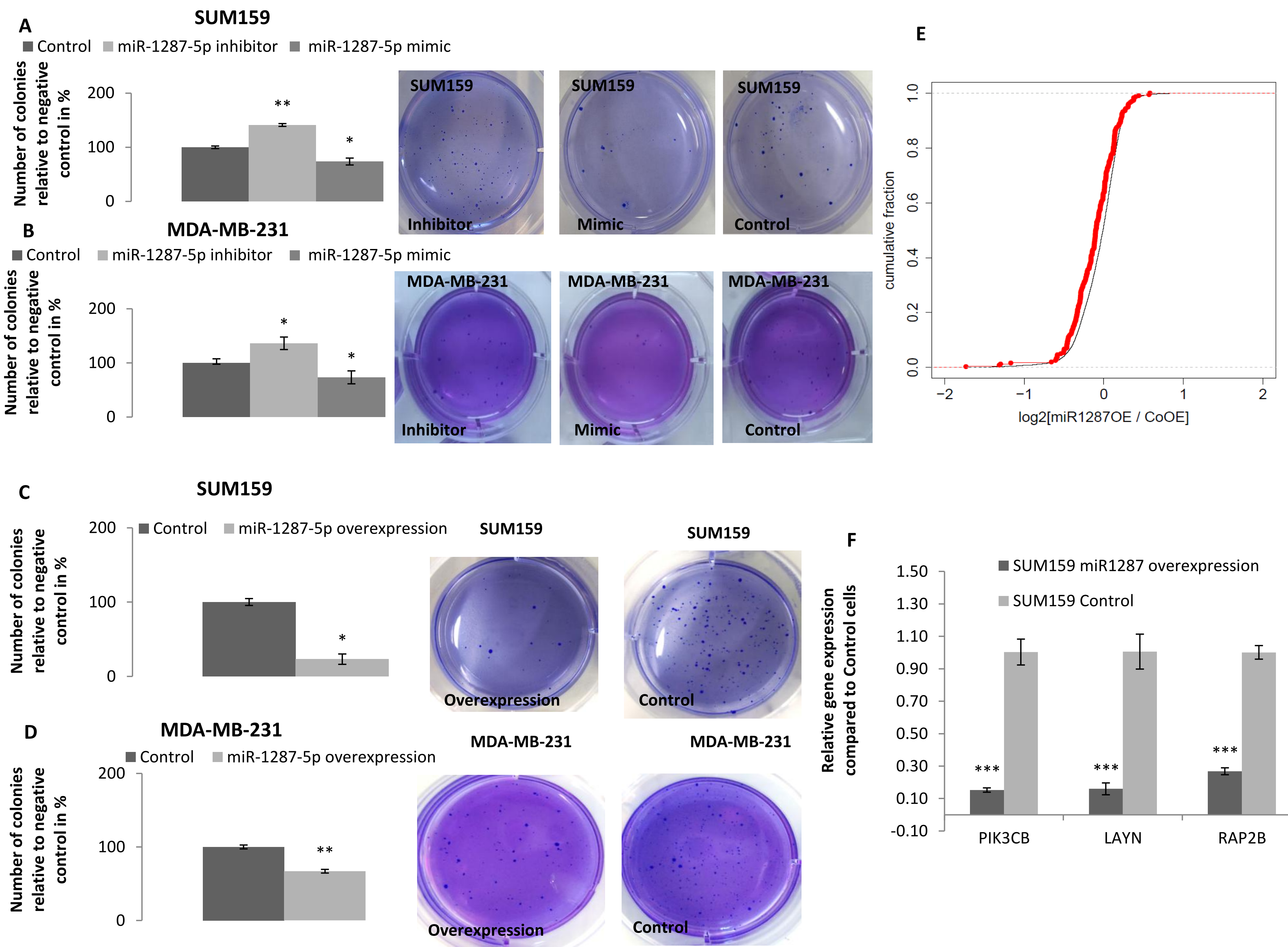

A
SUM159
Control
miR-1287-5p inhibitor
miR-1287-5p mimic
Number of colonies relative to negative control in %
200
100
0
**
*
SUM159
Inhibitor
SUM159
Mimic
SUM159
Control
B
MDA-MB-231
Control
miR-1287-5p inhibitor
miR-1287-5p mimic
Number of colonies relative to negative control in %
200
100
0
*
*
MDA-MB-231
Inhibitor
MDA-MB-231
Mimic
MDA-MB-231
Control
E
cumulative fraction
0.0
0.2
0.4
0.6
0.8
1.0
−2
−1
0
1
2
log2[miR1287OE / CoOE]
C
SUM159
Number of colonies relative to negative control in %
200
100
0
Control
miR-1287-5p overexpression
*
SUM159
Overexpression
SUM159
Control
D
MDA-MB-231
Control
miR-1287-5p overexpression
Number of colonies relative to negative control in %
200
100
0
**
MDA-MB-231
Overexpression
MDA-MB-231
Control
F
SUM159 miR1287 overexpression
SUM159 Control
Relative gene expression compared to Control cells
1.50
1.30
1.10
0.90
0.70
0.50
0.30
0.10
-0.10
***
***
***
PIK3CB
LAYN
RAP2B

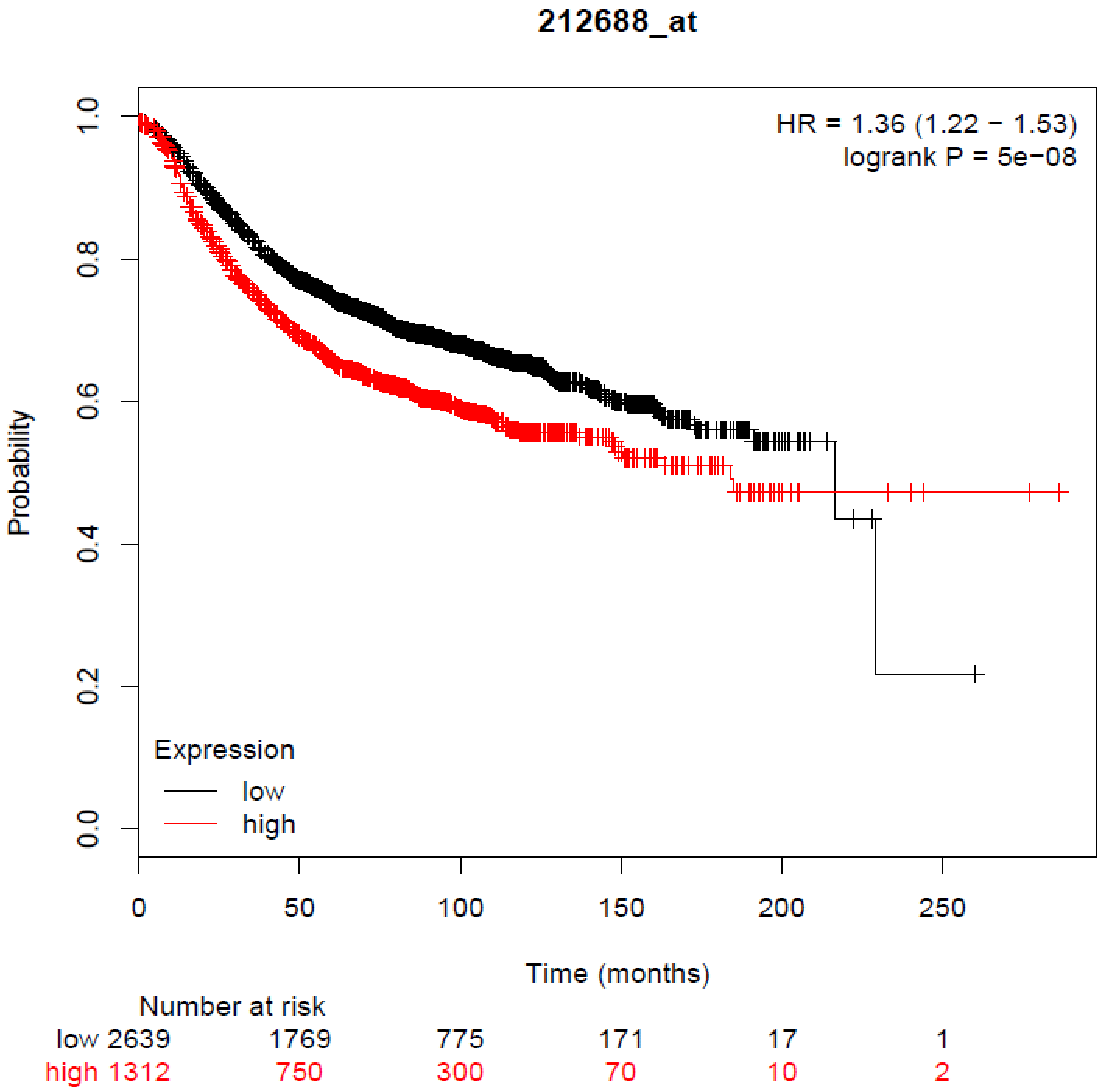
212688_at
HR = 1.36 (1.22 − 1.53)
logrank P = 5e−08
Probability
0.0
0.2
0.4
0.6
0.8
1.0
Expression
low
high
0
50
100
150
200
250
Time (months)
Number at risk
low 2639 1769 775 171 17 1
high 1312 750 300 70 10 2

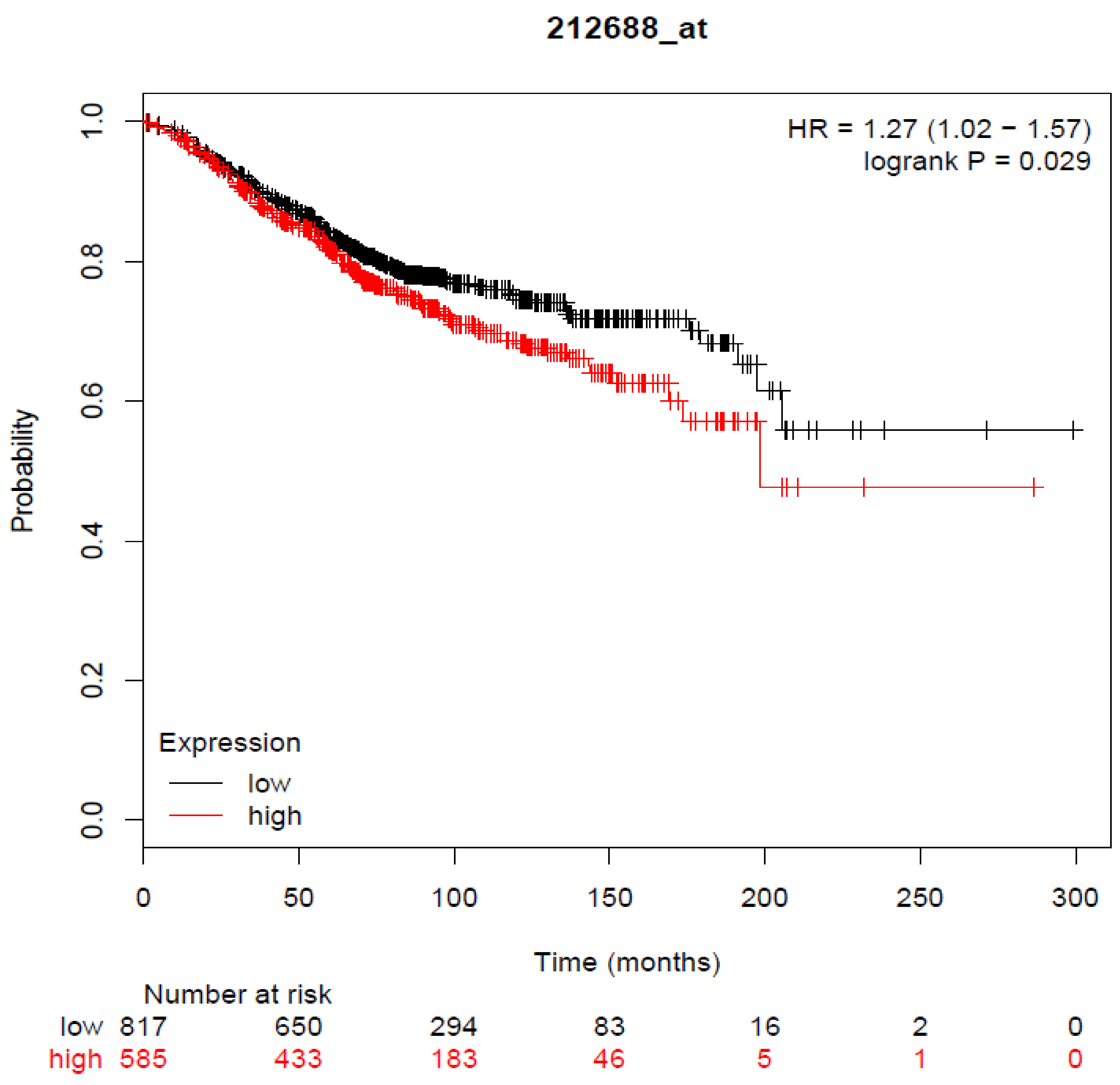
212688_at
HR = 1.27 (1.02 − 1.57)
logrank P = 0.029
Probability
1.0
0.8
0.6
0.4
0.2
0.0
Expression
low
high
0
50
100
150
200
250
300
Time (months)
Number at risk
low 817 650 294 83 16 2 0
high 585 433 183 46 5 1 0

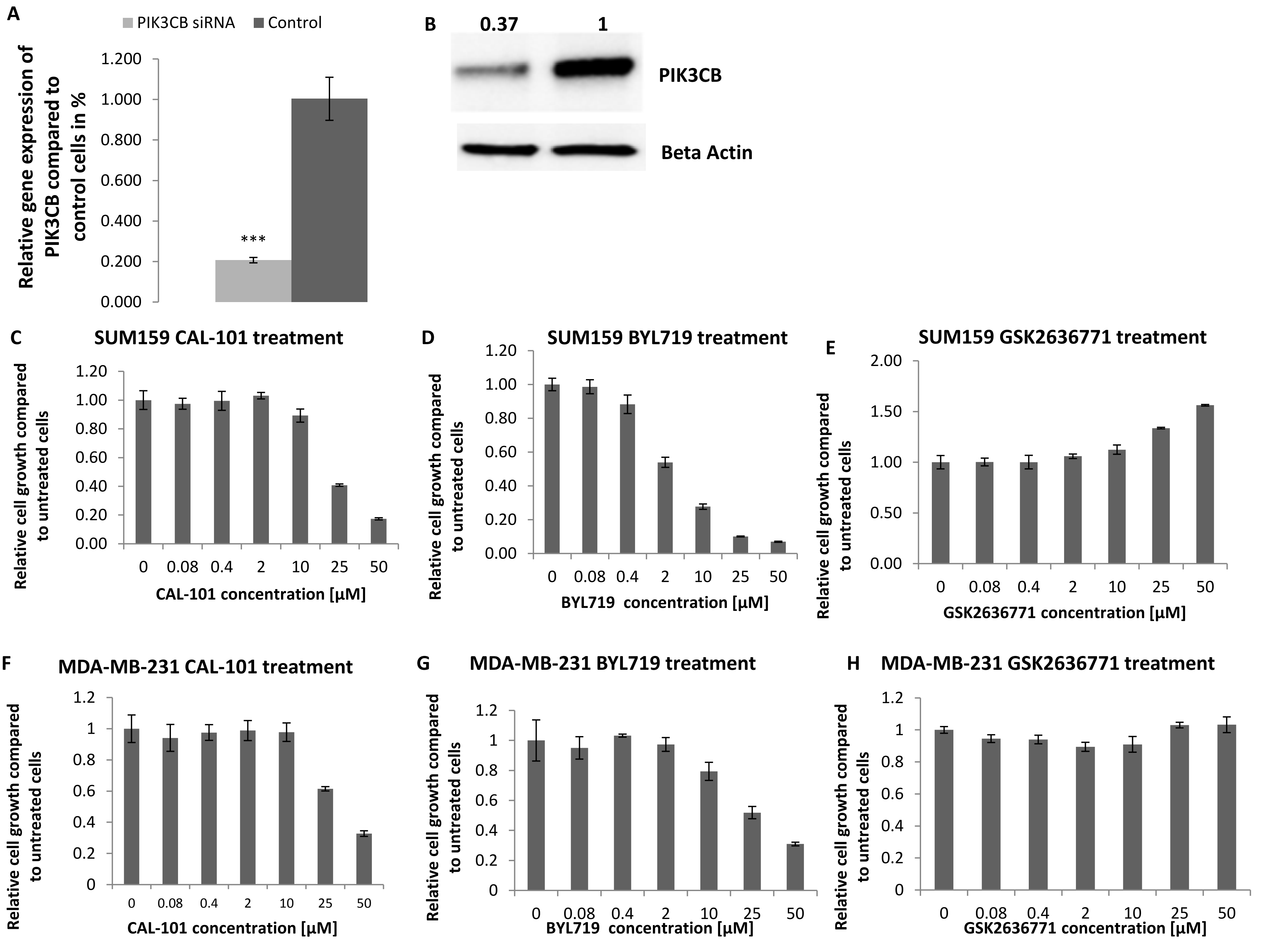

A
PIK3CB siRNA
Control
Relative gene expression of PIK3CB compared to control cells in %
1.200
1.000
0.800
0.600
0.400
0.200
0.000
***
B
0.37
1
PIK3CB
Beta Actin
C
SUM159 CAL-101 treatment
Relative cell growth compared to untreated cells
CAL-101 concentration [µM]
D
SUM159 BYL719 treatment
BYL719 concentration [µM]
E
SUM159 GSK2636771 treatment
GSK2636771 concentration [µM]
F
MDA-MB-231 CAL-101 treatment
CAL-101 concentration [µM]
G
MDA-MB-231 BYL719 treatment
BYL719 concentration [µM]
H
MDA-MB-231 GSK2636771 treatment
GSK2636771 concentration [µM]
0
0.08
0.4
2
10
25
50

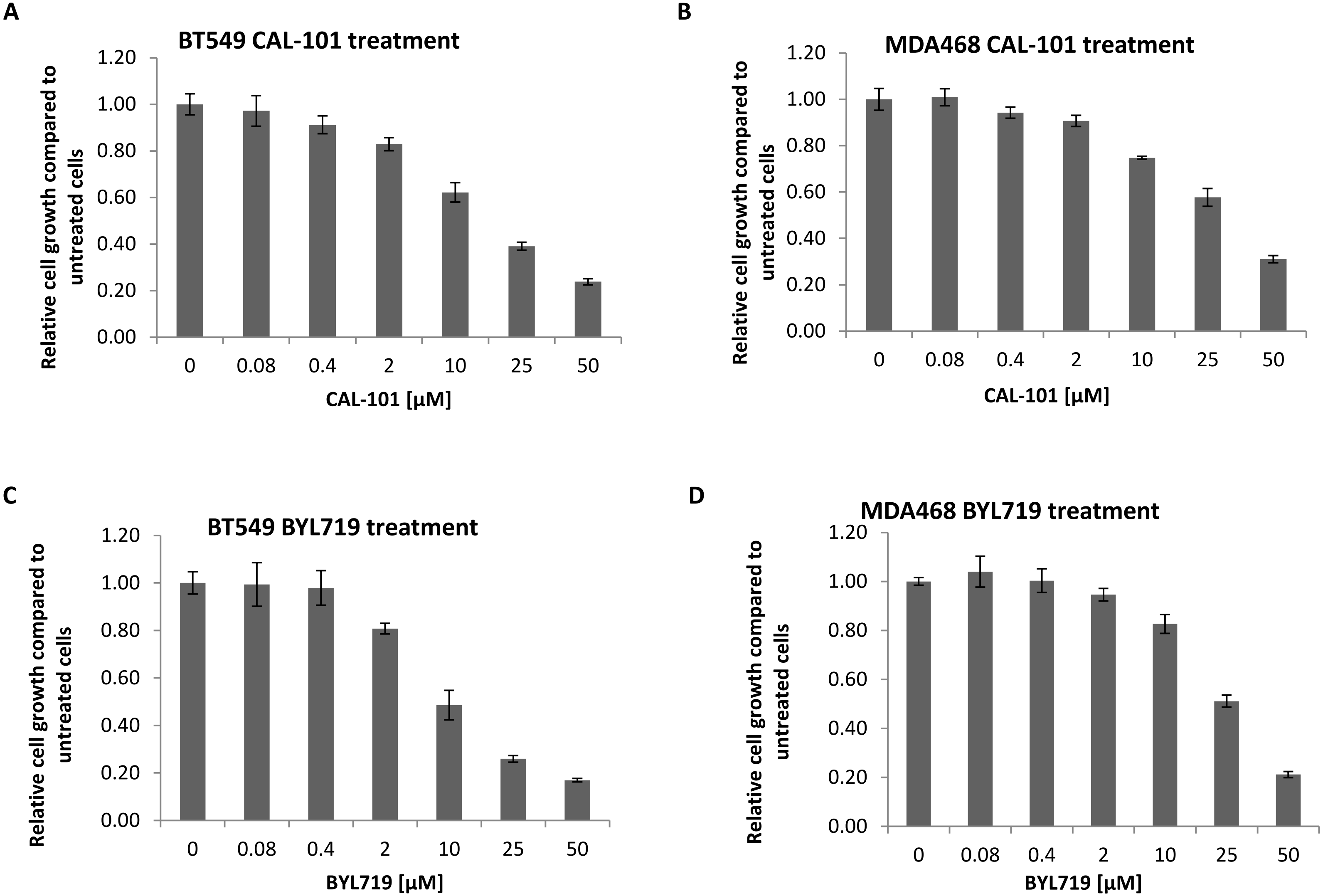
A
BT549 CAL-101 treatment
Relative cell growth compared to untreated cells
1.20
1.00
0.80
0.60
0.40
0.20
0.00
0
0.08
0.4
2
10
25
50
CAL-101 [µM]
B
MDA468 CAL-101 treatment
Relative cell growth compared to untreated cells
1.20
1.00
0.80
0.60
0.40
0.20
0.00
0
0.08
0.4
2
10
25
50
CAL-101 [µM]
C
BT549 BYL719 treatment
Relative cell growth compared to untreated cells
1.20
1.00
0.80
0.60
0.40
0.20
0.00
0
0.08
0.4
2
10
25
50
BYL719 [µM]
D
MDA468 BYL719 treatment
Relative cell growth compared to untreated cells
1.20
1.00
0.80
0.60
0.40
0.20
0.00
0
0.08
0.4
2
10
25
50
BYL719 [µM]
