# Supplementary material for: MiR-1287-5p inhibits triple negative breast cancer growth by interaction with phosphoinositide 3-kinase CB, thereby sensitizing cells for PI3Kinase inhibitors
Source: Breast Cancer Res. 2019 Feb 1;21:20. doi: 10.1186/s13058-019-1104-5 (PMC6359814; doi:10.1186/s13058-019-1104-5)
Supplement: Supplementary file 3 — Table S2. Downregulated mRNAs after miR-1287 overexpression in the cell line SUM159. This list shows 126 mRNAs that were at least 1.5-fold downregulated with a p-value of< 0.05. (DOCX 18 kb) [file 13058_2019_1104_MOESM3_ESM.docx]

Table S2: Downregulated genes after miR-1287 overexpression in the cell line SUM159. This list shows 126 mRNAs that were at least 1.5 fold –downregulated with a p-value of<0.05.

| Gene name | fold change |
| --- | --- |
| CPPED1 | **-5,04** |
| BEX1 | **-4,21** |
| SSX1 | **-3,08** |
| SSX2B | **-2,77** |
| SSX2B | **-2,77** |
| KDELC2 | **-2,76** |
| AC073236.3 | **-2,66** |
| TIMP3 | **-2,58** |
| CCND2 | **-2,56** |
| MIR205HG | **-2,55** |
| UGT8 | **-2,39** |
| PAGE2B | **-2,35** |
| RP3-410C9.2 | **-2,27** |
| XDH | **-2,23** |
| RNU6-1279P | **-2,19** |
| CASC21 | **-2,17** |
| RNY4P23 | **-2,17** |
| TP63 | **-2,15** |
| NEO1 | **-2,08** |
| DMKN | **-2,08** |
| FAR2P1 | **-2,08** |
| SDHAP2 | **-2,08** |
| CTD-2313J17.1 | **-2,07** |
| MUC13 | **-2,07** |
| MGP | **-2,05** |
| MAGEC1 | **-2,04** |
| RP11-382F24.2 | **-2,03** |
| LPAR3 | **-2,01** |
| LOC440173 | **-1,94** |
| CLDN1 | **-1,94** |
| APOC1P1 | **-1,89** |
| CA9 | **-1,88** |
| FBXO48 | **-1,87** |
| MAGEC2 | **-1,87** |
| RP11-11N9.4 | **-1,85** |
| RNA5SP201 | **-1,84** |
| CD274 | **-1,82** |
| RNF43 | **-1,82** |
| CRLF2 | **-1,81** |
| CADM1 | **-1,81** |
| CSRP2 | **-1,80** |
| MYLK | **-1,79** |
| MID1IP1-AS1 | **-1,78** |
| GPD2 | **-1,78** |
| PAGE2 | **-1,77** |
| RNU6-719P | **-1,77** |
| PIAS2 | **-1,77** |
| GS1-600G8.5 | **-1,77** |
| OCLN | **-1,77** |
| LOC441956 | **-1,76** |
| LOC441956 | **-1,76** |
| EXPH5 | **-1,75** |
| LOC344887 | **-1,75** |
| JAG2 | **-1,75** |
| NAP1L3 | **-1,74** |
| KIAA1324L | **-1,73** |
| RP11-30K9.4 | **-1,72** |
| CLHC1 | **-1,72** |
| KRTAP5-3 | **-1,71** |
| KRTAP2-3 | **-1,71** |
| CD24 | **-1,71** |
| TARSL2 | **-1,70** |
| RP11-129I19.2 | **-1,70** |
| NUPR1 | **-1,70** |
| GCSH | **-1,68** |
| RP11-706C16.7 | **-1,67** |
| PPP2R1B | **-1,67** |
| F3 | **-1,67** |
| COL5A2 | **-1,67** |
| ME2 | **-1,66** |
| NOV | **-1,64** |
| PSTPIP2 | **-1,64** |
| LOC101929484 | **-1,62** |
| FRZB | **-1,62** |
| ARRDC4 | **-1,61** |
| TMEM75 | **-1,61** |
| SPDYE3 | **-1,60** |
| HCLS1 | **-1,60** |
| CORO2A | **-1,60** |
| SPANXC | **-1,60** |
| MIR548AN | **-1,60** |
| SRGAP2-AS1 | **-1,60** |
| LRP4 | **-1,59** |
| MARVELD2 | **-1,59** |
| AC073842.19 | **-1,59** |
| TEC | **-1,59** |
| ZW10 | **-1,58** |
| DLL1 | **-1,58** |
| AC007050.17 | **-1,57** |
| ATF7IP2 | **-1,57** |
| LAYN | **-1,57** |
| PLD5 | **-1,56** |
| ZNF284 | **-1,56** |
| RNF168 | **-1,56** |
| LPPR4 | **-1,56** |
| ITGB3 | **-1,56** |
| SDHAP2 | **-1,56** |
| MIR1206 | **-1,56** |
| RP11-430B1.1 | **-1,56** |
| RP11-97O12.6 | **-1,55** |
| ZIC1 | **-1,55** |
| TIRAP | **-1,55** |
| RAP2B | **-1,55** |
| SLC27A2 | **-1,55** |
| INADL | **-1,54** |
| AC098973.2 | **-1,54** |
| RP11-706O15.7 | **-1,54** |
| RP11-307P5.1 | **-1,53** |
| RNA5SP463 | **-1,53** |
| RP11-626H12.2 | **-1,52** |
| SMAD2 | **-1,52** |
| POTEJ | **-1,52** |
| ASTE1 | **-1,52** |
| LRRC16A | **-1,52** |
| PCDHB6 | **-1,52** |
| ZNF559 | **-1,52** |
| ATMIN | **-1,52** |
| PIK3CB | **-1,51** |
| LOC101929988 | **-1,51** |
| ZNF136 | **-1,51** |
| RNU6-220P | **-1,51** |
| RNU6-678P | **-1,51** |
| HIST1H2AC | **-1,51** |
| CTD-2147F2.1 | **-1,51** |
| LOC100129617 | **-1,50** |
| F11R | **-1,50** |
